# A Review of Diagnostics Methodologies for Metal Additive Manufacturing Processes and Products

**DOI:** 10.3390/ma14174929

**Published:** 2021-08-30

**Authors:** Teng Yang, Sangram Mazumder, Yuqi Jin, Brian Squires, Mathew Sofield, Mangesh V. Pantawane, Narendra B. Dahotre, Arup Neogi

**Affiliations:** 1 Department of Materials Science and Engineering, University of North Texas, Denton, TX 76207, USA; Tengyang@my.unt.edu (T.Y.); SangramMazumder@my.unt.edu (S.M.); MangeshPantawane@my.unt.edu (M.V.P.); narendra.dahotre@unt.edu (N.B.D.); 2 Center for Agile and Adaptive Additive Manufacturing, University of North Texas, Denton, TX 76207, USA; yuqijin@my.unt.edu; 3 Department of Physics, University of North Texas, Denton, TX 76203, USA; BrianSquires@my.unt.edu (B.S.); Mathewsofield@my.unt.edu (M.S.)

**Keywords:** additive manufacturing, metal 3D printing, electron-based characterization, thermal imaging, ultrasonic test, ultrasonic evaluation, ultrasonic elastography, mechanical test, laser-induced breakdown spectroscopy, in-situ monitoring, ex-situ inspection, microstructure, defect

## Abstract

Additive manufacturing technologies based on metal are evolving into an essential advanced manufacturing tool for constructing prototypes and parts that can lead to complex structures, dissimilar metal-based structures that cannot be constructed using conventional metallurgical techniques. Unlike traditional manufacturing processes, the metal AM processes are unreliable due to variable process parameters and a lack of conventionally acceptable evaluation methods. A thorough understanding of various diagnostic techniques is essential to improve the quality of additively manufactured products and provide reliable feedback on the manufacturing processes for improving the quality of the products. This review summarizes and discusses various ex-situ inspections and in-situ monitoring methods, including electron-based methods, thermal methods, acoustic methods, laser breakdown, and mechanical methods, for metal additive manufacturing.


**Table of Contents**

1. Introduction: Metal Additive Manufacturing (AM) Technologies21.1 Fusion-Based AM Techniques21.2. Solid-State AM Techniques41.3. Mixed-Phase (Solid–Liquid) AM Techniques51.4. Surface Engineering via AM Techniques61.5. Comparison and Significance of AM versus Conventional Manufacturing72. Electron-Based Characterization and Diagnostics82.1 Electron Beam-Based Diagnosis/Characterization82.2. X-ray-Based Diagnosis/Characterization102.3. Electron–X-ray Combined Characterization Techniques103. Thermal Imaging133.1. Two-Wavelength Thermal Imaging under the Grey Body Approximation133.2. When the Grey Body Approximation Fails153.3. Discussion on Thermal Imaging164. Ultrasonic Inspection and Evaluation174.1. Ultrasonic Diagnostics174.2. Non-Destructive Ultrasonic Elasticity Measurement and Elastography244.3. Longitudinal and Transversal Sound Velocity Elasticity Measurement254.4. Comparison of Various Ultrasonic Techniques295. Mechanical Test305.1. Elasticity Test with Information on Plasticity305.2. Mechanical Test Provides Only Plasticity355.3. Impact Test375.4. Fatigue Test385.5. Advantages and Disadvantages, Comparison and Discussion of Mechanical Testing396. Laser-Induced Breakdown Spectroscopy397. Other Diagnostic Methods418. Conclusions and Discussion42Reference43

## 1. Introduction: Metal Additive Manufacturing (AM) Technologies

Additive manufacturing (AM) paves the way towards the next industrial revolution as it offers an impressive set of advantageous characteristics over traditional manufacturing. The progress of AM is primarily driven by its flexibility to fabricate components through computationally aided models in a layer-by-layer fashion [1]. By its nature, this technique is in contrast with the traditional subtractive/formative manufacturing techniques. Most relevant AM techniques commonly use a powder/wire/sheet as a precursor material, consolidated layer-by-layer to fabricate a part [2,3]. AM has attracted much attention for over a decade due to its inherent advantages, which eliminate significant constraints that hinder optimal design, material, and cost efficiency, and the ease of manufacturing complex parts [4,5]. AM techniques have already been around for more than 20 years, but, for a long time, they had been restricted to the rapid manufacturing of porous structures and prototypes [6].

Nonetheless, with gradual and substantial progress in technology, both part density and the quality of products additively manufactured have substantially improved. Thus, this has led to the evolution of its first application in tool inserts with conformal cooling [7] and medical applications, e.g., as dental prostheses [8]. The reliable manufacturing of dense parts for several materials, including, but not limited to, steel, titanium, and aluminum, is feasible using certain AM fabrication/printing techniques [9].

**Figure 1:**
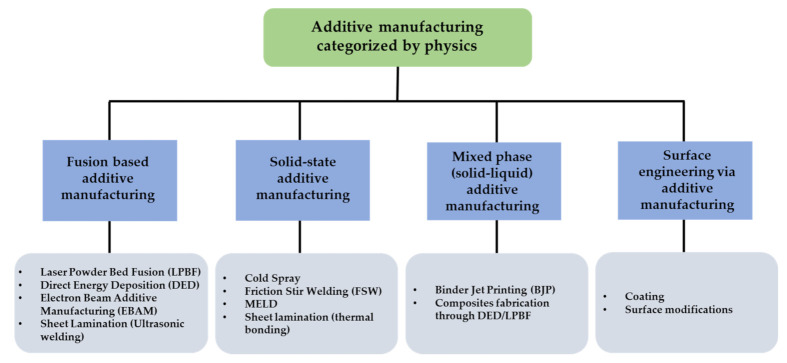
Additive manufacturing techniques are categorized by the physics involved during their fabrication.

**Figure 2:**
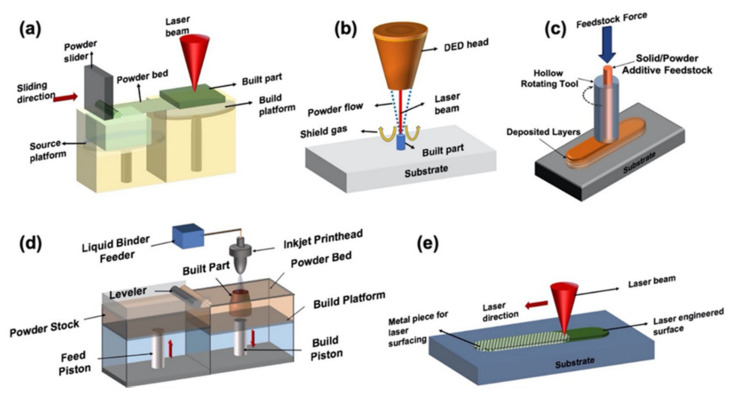
The schematic diagram for (**a**) LPBF, (**b**) DED fusion bed AM processing, (**c**) friction stir AM solid-state processing, (**d**) binder jet printing mixed-phase AM processing and, (**e**) laser surface engineering via AM.

AM is revolutionizing more and more from rapid prototyping to agile manufacturing applications [10]. It requires in-depth insight into the process and the microstructural features as an outcome of the process parameters, and it renders unique properties to the additively printed parts. Various AM processes for metals are based on the fundamental concept of layer-wise material deposition. However, each processing technique has unique and distinct physical phenomena due to the energy source, operating conditions, precursor materials, and mechanisms involved in metallurgical consolidation [11]. During the metallic AM process, the materials in use encounter complex thermokinetics that involve a combination of directional heat extraction, molten pool dynamics, rapid solidification, and repeated cycles of heating and cooling. These factors influence the physical integrity of the AM component and microstructure with peculiar properties different from conventionally processed parts [12]. All these aspects affect the mechanical and chemical properties of the printed components. Thus, it is essential to recognize the categorization of different AM processes based on the physics associated with each AM process during the fabrication of a component (Figure 1 and Figure 2).

### 1.1. Fusion-Based AM Techniques

Fusion-based AM processes (Table 1) involve the complete melting of metal precursors (powder, wire, sheet) followed by solidification. A beam of a heat source (laser or electron) with controlled spot size, power, scanning speed, and pattern (spatial energy distribution) is used to melt the metal precursor material, which fuses and solidifies once the beam travels away. Upon interaction of the heat source with the precursor, the precursor’s energy is absorbed, leading to melting and consolidation with the previous layer based on the interaction volume.

Because of the rapid cooling rates, the fusion-based AM process gives rise to a complex non-equilibrium microstructure, which imparts unique properties to the printed component. Laser powder bed fusion (LPBF), also known as selective laser melting (SLM), is a well-known fusion-based AM technique [13,26], where powder particles are spread on a substrate (seed plate) to form a powder bed. A rastering laser beam is allowed to scan the powder bed in a defined scanning pattern. Direct metal deposition (DMD) [27], also known as direct energy deposition (DED) [14], is another common fusion-based AM processing technique. In DED or DMD, the precursors (powder or wire) are transferred directly through a nozzle to the melt pool created by a rastering laser beam on the surface of the substrate. In DED, the laser power is kept higher, and the laser spot size is larger, giving rise to a higher deposition rate than the LPBF process. Fabrication of a mechanically sound component requires an optimized laser scanning speed in DED that varies between 5 and 20 mm/s. In LPBF, it is consolidated at a relatively higher scanning range of 200–1200 mm/s. Hence, the fluid dynamics associated with these processes are significantly different, influencing the evolution of microstructure/phase and the mechanical integrity of the components built by these processes. The thermokinetics involved in these two processes differ significantly, leading to a difference in the thermodynamic states of the resultant phases within the products fabricated through the different melt fusion-based AM processes. 

### 1.2. Solid-State AM Techniques

In the solid-state AM process (SSAM), the material does not melt. Instead, the layers are joined in the solid state under accelerated diffusion, forged consolidation through high friction, pressure, and impact. Since there is no melting in SSAM, the complex fluid dynamics associated with fusion-based AM techniques are absent. In turn, this is beneficial in minimizing the chances of porosity and physical defect formation associated with fusion-based AM in the printed components. Some of the SSAM techniques (Table 2) include cold spraying [15] and friction stir welding [16]. Cold spraying is considered the most common SSAM technique. In it, supersonic velocities are imparted to metal particles by placing them in high-pressure, heated air, an inert gas stream that is expanded via a convergent–divergent nozzle. High pressure and temperature result in high gas velocities, yielding high particle acceleration within the gas stream. The particles entrapped in the gas stream are bombarded towards a surface embedded on impact, forming a strong bond. Subsequent spray passes cause increments in structure thickness, and the adhesion of the metal powder to the substrate and the cohesion of the deposited material is achieved in solid state [17].

The concept of friction stir welding (FSW) and friction stir processing (FSP) was eventually developed as a generic tool for microstructural modifications based on the basic mechanism of FSW [18]. In this process, a rotating tool is inserted into a monolithic workpiece for localized microstructural modification, leading to specific property tuning. MELDTM is an emerging SSAM technique based on the concept of FSW [19]. Within this method, the filler material is delivered through a rotating, hollow tool. Dynamic contact friction generates plastic deformation during the processing, leading to solid-state bonding between material and substrate [20]. MELDTM can be used to additively manufacture a wide range of metals and metal matrix composites (MMCs) with low residual stress and increased density, with significantly lower energy consumption than the conventional fusion-based AM processes. A certain category of sheet lamination known as thermal bonding is also an SSAM technique based on applying thermal energy to accelerate diffusion and result in bonding between two sheets [21]. The sheet lamination-based additive manufacturing involving ultrasonic welding of the sheets through mechanical vibration produced by the sonotrode can also be categorized under solid-state AM techniques.

### 1.3. Mixed-Phase (Solid–Liquid) AM Techniques

Mixed-phase AM techniques are those in which the building components exist in both solid and liquid phases, which requires building components either melted using a heat source and altering the processing parameters or using them both solid and liquid phases. In AM processes where at least one component needs to be melted, the process is similar to the fusion-based AM processes discussed above. A heat source (laser/electron beam) is applied to the precursor material to melt it. However, in mixed-phase AM techniques, the total melting of the precursor material system is not intended. Rather, partial or complete melting of some selective components of the precursor mixture is achieved. Such AM processing techniques are the ones used for printing alloys or even composites. There is a continuous matrix phase impregnated with a reinforcing dispersed phase, and often, metal is the matrix and ceramic is the reinforcing phase [22,23,24]. Apart from alloy and composite formation, LPBF, DED, and EBM processes where intentional incomplete powder melting is attempted by selective alteration in processing parameters can also be categorized as mixed-phase AM processing. Hence, this variation of a mixed phase in which components are melted before printing using a heat source also involves the laser- and electron-based processing techniques described in the fusion-based AM techniques. A comparison is provided in Table 3.

Binder jet printing (BJP) is also a very commonly used mixed-phase AM technique [25,28], usually utilizing solid-phase powder(s) with a liquid-phase binder, and is a standalone process variation in the mixed-phase AM processing category. BJP uses an inkjet printing head to deposit liquid binder on top of the metal powder selectively. Eventually, the binder is left to dry out, and a fragile binder–metal mix, also known as the “green body”, is formed. The subsequent steps include curing and sintering this “green body”, which renders mechanical strength to the printed component. Infiltration by a second material is achieved by introducing the sintered part with a different material having a lower melting point when compared to that of the sintered part. The system is then sufficiently heated to achieve complete melting of the second material, which, as a result, infiltrates the sintered part, giving rise to a denser composite with unique properties. 

### 1.4. Surface Engineering via AM Techniques

Surface engineering involves the total melting and consolidation of metal precursors on the top of a previously fabricated component. The techniques deal with surface modifications of the already built part, which is the fundamental difference between this category of AM techniques and the others mentioned previously. These surface modifications might range from altering surface roughness to additively depositing a coating on it to achieve unique properties and facilitate the use of the built part for desired purposes. Such an approach allows the fabrication of high volumes of components using conventional manufacturing techniques and inexpensive materials followed by the fabrication of surface regions with expensive and high-performing materials with near neat dimensions.

Surface engineering via AM (SEAM) is a concept for the in-situ synthesis of advanced materials by complex motion systems integrated with various sensors for accuracy and remote operations. Site-specific in-situ material synthesis and fabrication of this material into a desired shape/form are the two processes simultaneously facilitated by SEAM. Hence, the uniqueness of SEAM places it in contrast to traditional AM techniques; this method provides significant savings in materials and processing time [38]. A converging laser beam is an incident that melts a liquid pool on the surface of a metal substrate and is moved relative to the beam direction. Powder particles are blown from a fixed nozzle into the melt pool and incorporated on the surface as the pool’s trailing end cools and solidifies. Surface coating involves the consolidation and solidification of one or more layers of a different metal on a built part. The complex fluid dynamics associated with the laser melt pool will also depend on the affinity of the two dissimilar materials. Apart from rendering unique thermokinetics and dynamics to the process, this might also be a governing factor in the bead diameter of the coating layer/s. An application-oriented advanced manufacturing system-based approach primarily drives SEAM applications. Previously published work on SEAM includes synthesizing a composite coating with the formation of transition metal intermetallic via extending the solid solubility of tungsten in an aluminum substrate using laser-based SEAM. Moreover, a high-entropy alloy (HEA) coating from a mixture of elemental powders for AlCoCrFeNi and AlCrFeNiTa with direct laser deposition (DLD) was successfully achieved [39,40].

### 1.5. Comparison and Significance of AM versus Conventional Manufacturing

There are several benefits AM possessing over the traditional manufacturing methods, including, but not limited, to cost and material efficiency, overcoming the critical component design aspects, speed, innovation, transformation, quality, and impact [41]. AM is truly innovative as it leads to new opportunities and lends itself to numerous possibilities to enhance manufacturing efficiency. AM significantly streamlines traditional subtractive methods and carries the potential (Table 4) to rise as the norm in the near future. Technical limitations restrict each manufacturing method’s application in processing a specific material, complex geometries, or repressive production costs. 

On the contrary, conventional machining enables the production of precision components, but the complexity level is compromised [42]. The convenience of 3D printing can enable the consumer to become their micro-manufacturers; simply downloading a 3D printing file can create their shapes, potentially reducing the need for logistics, as designs can be transferred electronically. Thus, the decentralization of manufacturing can be achieved. Researchers have also investigated the environmental aspects of both the additive and subtractive manufacturing processes [43]. A recent study has proposed a combined indicator for environmental impact ratio and volume of material removal ratio [29]. By way of illustration, from the study, electron beam melting (EBM) is eco-friendlier or “green” and a good manufacturing choice for components with a complex shape that otherwise requires substantial material removal with subtractive manufacturing methods. While manufacturing the component, energy consumption by EBM and milling is virtually identical; however, the key player in terms of environmental impact is the production of powder for EBM and the production and recycling of chips for milling. Thus, the advent of AM in the manufacturing industry will lead to more fluid product developments with reduced environmental impact.

## 2. Electron-Based Characterization and Diagnostics

**Figure 3:**
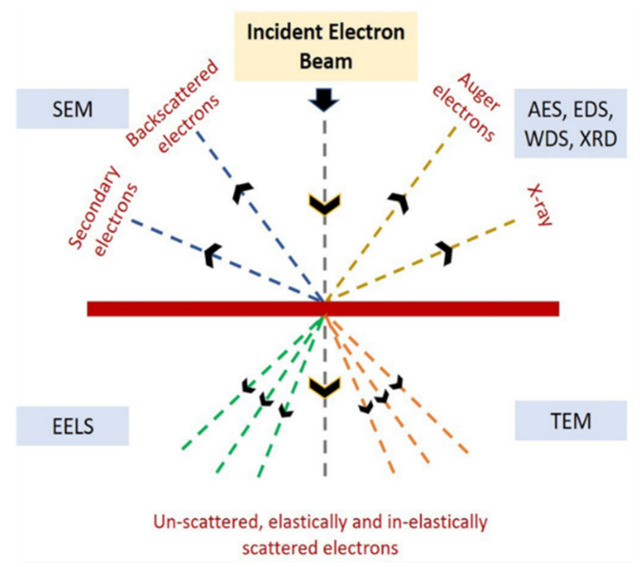
Various interaction products during an incident electron beam and a sample.

Electrons accelerated onto a material leads to a variety of radiative products due to the interaction between the electron beam and the atoms within or on the material. These radiative species and the typical signals used for imaging and quantitative and semi-quantitative analysis (as shown in Figure 3 and Table 5) might include, but are not restricted to, secondary or reflected (backscattered) electrons, X-rays, photoelectrons, and visible light (cathodoluminescence). Accelerated electrons might pass through the sample without (or minimal) interaction and undergo elastic or inelastic scattering. When an electron beam impinges on a sample, electron scattering, photon, and X-ray production develop in a volume that depends on the acceleration potential of the incident beam and the interaction volume. Likewise, the interaction between X-rays and material also forms the basis of several material characterization techniques. When X-rays are incident on a material, part of the radiation is absorbed, and the rest are scattered. If neither of these occurs, the X-rays are transmitted through the material. Absorption of X-rays results in fluorescence and is the basis of several characterization techniques in materials. The several electron- and X-ray-based characterization techniques are categorized as discussed below.

### 2.1. Electron Beam-Based Diagnosis/Characterization

Scanning electron microscopy (SEM) is a material characterization technique that involves a beam of electrons scanning the surface of a sample and producing images demonstrating the sample’s microstructure (topography and composition). Among the various signals produced due to the sample and the electron beam interaction, secondary electrons (SE) and backscattered electrons (BSE) are the two signals of interest in SEM, which are detected and studied for sample characterization. Owing to their low energy (<50 eV), SE originates from within a few nanometers below the sample surface [49]. The angle of incidence of the beam to the sample surface is directly proportional to the interaction volume, which controls the amount of SE being ejected. Hence, steep surfaces and edges tend to appear brighter than flat surfaces. BSE is the high-energy electrons backscattered out of the specimen during electron beam and sample interaction events. Elements with a high atomic number backscatter electrons more strongly and appear brighter than elements with a low atomic number. Therefore, BSE conveys information about different chemical compositions [50].

Transmission electron microscopy (TEM) refers to a microscopic characterization technique for materials wherein a beam of electrons is transmitted through a sample to form an image. The transmission of the electron beam through the sample is facilitated by the ultra-thin section (<10 nm in thickness) of the sample [51]. The image produced from the interaction of electrons with the sample as the beam traverses through it is magnified and focused onto an imaging device. Owing to the much shorter wavelength size of the electrons, the optimal resolution attainable for TEM images is many orders of magnitude higher. Hence, TEM can reveal the finest possible details in the internal structure of a material.

Electron energy-loss spectroscopy (EELS) is another characterization technique for materials that depends on a coherent electron beam’s interaction with a sample [52]. A beam of electrons may be inelastically scattered due to its interaction with a sample, which results in the electron beam’s energy loss, and is bent through a small angle (5–10 milliradians). The energy distribution plot of all the inelastically scattered electrons gives information about the sample’s local environment where the electron beam is incident. Hence, this relates to the physical and chemical properties of the sample. EELS is used to determine atomic compositions, surface properties, chemical bonding, and element-specific pair distance distribution functions in materials [30].

Auger electron spectroscopy (AES) is performed based on several interactions resulting from an incident electron beam on a material surface. This spectroscopy technique is based on the Auger effect [31] and is used to analyze the chemical composition of the surface of a material by measuring Auger electron energies. The surface sensitivity in AES occurs because the emitted electrons usually are in the energy range of 4–50 keV. At these values, electrons have a short mean free path in a solid. Hence, the escape depth of the electrons is localized to a few nanometers of the target surface, which imparts extreme sensitivity to the surface species [32]. Scanning Auger microscopes (SAMs) can produce high-resolution, spatially resolved chemical images. An intensity map correlates to grayscale on a monitor, with white areas corresponding to higher element concentrations. Sputtering is sometimes used with AES to perform depth profiling experiments. Sputtering helps to remove the thin outer layers of the surface, and hence, AES can be used to determine the underlying compositions [33].

**Table 5:** Summary of electron-based material characterization techniques [53,54,55,56,57,58,59].

Electron-Based Analysis Method	Working Principle	Measuring Process	Measuring Characteristics
Scanning Electron Microscopy (TEM)	Detecting secondary and backscattered electrons resulting from sample and electron beam interactions	Electrons in range of 1–30 kV are applied to the sample, and secondary, backscattered electrons are detected	Measuring fine surface morphology and phase differentiation. Observing cross-section of samples. Resolving power can be ~1 nm
Transmission Electron Microscopy (TEM)	Primary electron traverse through sample, producing both transmitted and diffracted electron beam	Transmission of electrons in range of 100–1000 kV through a sample and detection of absorption and diffracted electrons by the interactions between atoms	Fine crystal defects, grain size, lattice structure. Resolving power can be around 2 nm
Electron Energy Loss Spectroscopy (EELS)	Electron beam–sample interactions result in inelastic scattering of electrons	A coherent electron beam incident on a sample produces inelastically scattered electrons having lesser energy. These inelastically scattered electrons are measured.	Atomic compositions, surface properties, chemical bonding in sample
Auger Electron Spectroscopy (AES)	Primary electrons incident on the sample surface and resulting in Auger electrons	Auger electrons, unique to each atom and resulting from electron beam and sample interaction, are measured	Composition analysis and impurities detection

### 2.2. X-ray-Based Diagnosis/Characterization

X-ray diffraction (XRD) is one of the most widely preferred characterization techniques to identify unknown crystalline materials [34]. XRD is based on the constructive interference of monochromatic X-rays and a crystalline sample. When the conditions satisfy Bragg’s law nλ=2dsinθ,  the interaction of the X-rays and the sample results in constructive interference. The intensity of diffracted X-rays is continuously recorded as the sample, and the detector rotates through their respective angles. An intense peak is seen to rise when the material contains lattice planes with d-spacing appropriate to diffract X-rays at that value of θ. Results are presented as peak positions at 2θ values and X-ray counts (intensity) in an X-Y plot or a table. The d-spacing of each peak is calculated by solving Bragg’s equation for appropriate values of λ. Upon determination of the d-spacing value, it is compared with that of the known materials for identification.

X-ray tomography (XRTM) is a well-known technique for characterizing materials in three dimensions (3D) [35]. It has been employed to study materials that demonstrate large differences in absorption (often related to density) between the microstructural and the host matrix characteristics. For example, XRTM studies have been performed to detect voids within metal matrices and inclusions of other secondary phases within metals [36].

### 2.3. Electron–X-ray Combined Characterization Techniques

Electron backscattered diffraction (EBSD) (Table 6) is a material characterization technique that combines both electrons and X-rays in conveying information about the crystalline orientation, structure, strain, or phase in the material [37]. EBSD can be conducted in an SEM equipped with a phosphor screen. In SEM analysis, the reflected electrons or BSE may be scattered at a Bragg angle and diffracted to form Kikuchi bands, corresponding to lattice diffracting crystalline planes. Each band can then be indexed individually by the Miller indices of the diffracting plane, which has resulted in it. These diffracted electrons collide with the phosphor screen located within the sample chamber of the SEM at an angle of approximately 90°, which causes fluorescence of the phosphor. Above is the basic principle of EBSD, and this technique is widely used in the materials characterization field.

Energy-dispersive X-ray spectroscopy (EDS or EDX), also known as energy-dispersive X-ray analysis (EDXA), can be categorized as one of the electron and X-ray combined material characterization techniques used for the elemental analysis or chemical characterization of a sample material [60]. In this technique, an electron beam (10–20 keV) strikes the sample’s surface, and as a result, X-rays are emitted from the irradiated sample. EDX/EDS is not a surface characterization technique as X-rays are generated in a region around 2 microns in depth. A 2D image of each element in the sample can be acquired by moving the electron beam across the sample.

Wavelength dispersive spectroscopy (WDS) (Table 6) is another material characterization technique involving an electron beam interaction with the sample surface and resulting in the emission of X-rays from it [61]. These emitted X-rays are energy-specific to the element that results in its emission. When the X-rays enter the WDS, they hit a crystal with defined lattice parameters. This results in the diffraction of the X-rays, and the amount of this diffraction depends on their energy. The instrument is generally equipped with multiple diffractions to cover the entire energy range of interest. WDS is used for separating EDX peak overlaps, trace element identification, and elemental quantification. Applications include determining alloy composition, mapping second phases in certain alloys, and defect identification [62].

X-ray photoelectron spectroscopy (XPS) (Table 6) is a surface-sensitive quantitative spectroscopic technique that measures the elemental composition in parts per thousand (ppm) range. It uses an electron beam to emit X-rays from the samples. XPS can also be used to determine the empirical formula, chemical state, and electronic state of the elemental constituents in the sample. XPS is particularly useful as it identifies the elements within the sample and the other elements bonded to it. Hence, if a sample has metal oxide, XPS can also detect the valence state of the metal element [44]. In this technique, the surface of the sample is irradiated with an X-ray having an energy h*ν*. Photons that are mono-energetic knock out an electron from atoms in the surface region, whereas photons with higher energy (h*ν*) can penetrate deeper into the sample surface. A spectrum is obtained by measuring the characteristics of the electrons generated from the sample surface [45].

**Figure 4:**
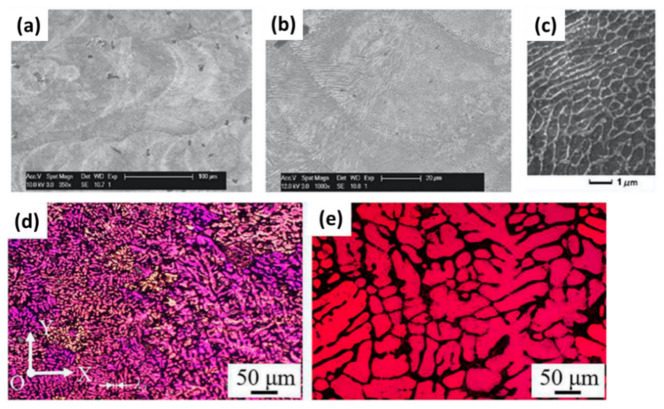
SEM images of LBM-printed (**a**–**c**) 18 Ni-300 steel showing finer grains [53]. (**d**,**e**) The grain size of the as-deposited and as-cast ALSiMg0.6 [64].

Generally, an evolution in microstructural features is accompanied by AM, especially in the laser-based or electron-based processing techniques, where the materials are subjected to complex thermal stress cycles [46,47]. This thermal stress cycle occurs due to rapid heating of the material above its melting temperature, followed by rapid cooling as soon as the heat source moves away, and is responsible for the distinctly different microstructures in such AM-processed materials compared to their conventionally manufactured counterparts. These microstructural evolutions are significant as they govern several material properties. Electron-based, X-ray-based, and a combination of electron- and X-ray-based characterization techniques are the basis of characterization in additively printed metals. As discussed above, the evolved microstructural aspects, such as grains within AM-printed, samples compared to their conventionally manufactured counterparts, are always visually confirmed by observing the AM-printed parts under SEM [48,63]. As an example, Kempen et al. [53] observed fine-grained microstructures in LBM-fabricated maraging steel (18-Ni300) in its as-fabricated state (Figure 4a–c). A similar observation may include a report by Yang et al. [64], where fine-grained structures were observed in the as-deposited AlSiMg0.6, compared to the wrought sample [65] (Figure 4d,e).

**Figure 5:**
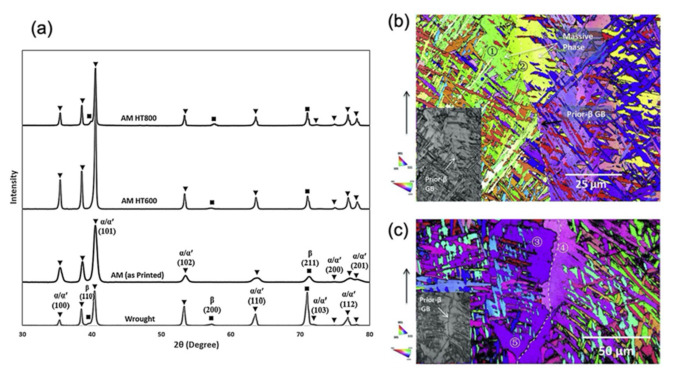
(**a**) XRD data for both AM and wrought Ti6Al4V indicates the sole presence of α’ martensite within AM samples [59]. (**b**,**c**) EBSD color inverse pole from figures of EBM-printed Ti6Al4V samples [66].

Furthermore, governed by the thermodynamic free energy content principles, changes in phases, sometimes massively, are often found in AM-printed samples. Researchers worldwide have been using X-ray-based techniques, especially XRD, to identify these changes in phase content within AM-printed metals compared to their conventionally manufactured counterparts [55,56]. Ettefagh et al. [57] performed a comparative study on the phase content of additively printed and conventionally manufactured Ti6Al4V and reported the sole presence of martensite in SLM-processed Ti6Al4V, which was distinctly different and absent in the counterparts processed conventionally (Figure 5a). Apart from these, combined electron- and X-ray-based techniques, especially EBSD and EDX, have gained massive interest as these characterization techniques help to elucidate the crystal structure and orientation. Moreover, elemental content within AM-printed parts varies from the conventionally manufactured process. It has important information with which to understand and differentiate the distinct and peculiar mechanical and material characteristics exhibited by metals processed by additive and subtractive manufacturing [58,59]. Lu et al. [66] observed and reported massive transformation in EBM-printed Ti6Al4V, where EBSD color inverse pole figures (IPFs) were shown to demonstrate the grain boundary-crossing phenomenon in the samples (Figure 5b,c).

## 3. Thermal Imaging

### 3.1. Two-Wavelength Thermal Imaging under the Grey Body Approximation

**Figure 6:**
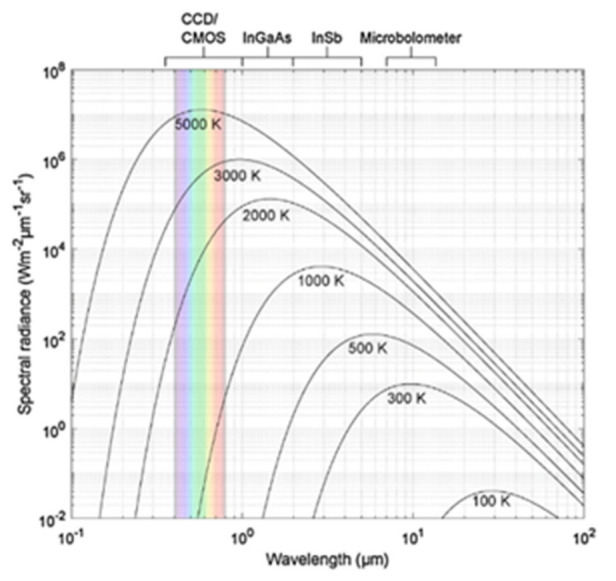
Spectral radiance of a blackbody at various temperatures. Wavelength sensitivity ranges for various detectors are indicated above [67].

Objects with a non-zero temperature emit electromagnetic radiation in accordance with Planck’s law of blackbody radiation:

(1) $$B\left(\lambda ,T\right)=\frac{2hc^{2}}{\lambda^{5}}{\left(exp\left(\frac{hc}{k_{B}\lambda T}\right)-1\right)}^{-1}$$

where $B\left(\lambda ,T\right)$ is the spectral radiance as a function of wavelength (λ) and temperature (*T*), h is Planck’s constant, c is the speed of light in a vacuum, and $k_{B}$ is Boltzmann’s constant. Figure 6 illustrates the spectral dependence of blackbody emission as a function of temperature.

The emissivity (ϵ) of real materials is rarely that of a true blackbody, and the optical path transmissivity (σ) is not identical to that of a vacuum [67]. As such, Equation (1) must be modified as follows:

(2) $$B’\left(\lambda ,T,\;\epsilon ,\sigma \right)=\frac{2hc^{2}\epsilon \sigma }{\lambda^{5}}{\left(exp\left(\frac{hc}{k_{B}\lambda T}\right)-1\right)}^{-1}$$

where ϵ and σ range from 0 to 1 and are functions of the material, environment, wavelength, and temperature. This problem of unknown coefficients may be overcome by measuring the ratio of intensities of two nearby wavelengths $\left(\lambda_{1},\lambda_{2}\right)$, where the approximation $\epsilon_{1}\approx \epsilon_{2}$ (grey body approximation) is valid. As such, the ratio of emission intensity values at the two wavelengths is

(3) $$\frac{I_{1}}{I_{2}}\approx \frac{A_{1}\epsilon_{1}\lambda_{2}^{5}}{A_{2}\epsilon_{2}\lambda_{1}^{5}}exp\left(\frac{hc}{k_{B}T}\left(\frac{1}{\lambda_{2}}-{\frac{1}{\lambda }}_{1}\right)\right)$$

where $A_{1},A_{2}$ are the combined optical path transmissivities and detector sensitivities at the two wavelengths, and the approximation $\frac{hc}{\lambda }\gg k_{b}T$ (Wien approximation) has been applied for simplification. The coefficients $A_{1},A_{2}$ may be obtained empirically, and thus a calibration curve may be generated for the ratio $\frac{I_{1}}{I_{2}}$ that does not depend strongly on emissivity [67]. This approximation only holds if the emissivity variance is negligible with respect to all other parameters besides wavelength and temperature (structure, composition, local phase, etc.).

**Figure 7:**
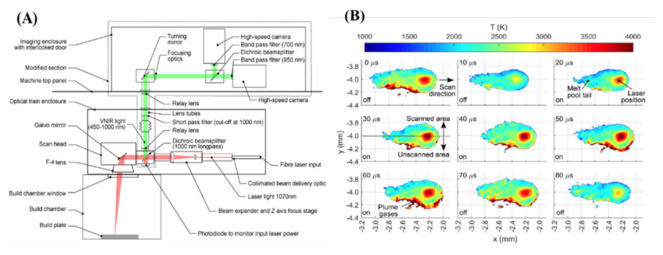
Schematic of the optical path (**A**) and representative temperature maps (**B**) of a high-speed two-wavelength pyrometric imaging apparatus [67].

Furumoto et al. demonstrated the use of two-color pyrometry to measure the temperature of a single point of the melt pool while simultaneously visualizing the process with a high-speed camera [68]. More compellingly, Hooper designed and implemented a two-color pyrometry apparatus that uses two high-speed cameras to monitor separate wavelengths simultaneously [67]. In this way, he measured the spatial and temporal variation in the thermal signature of a melt pool with a 100 kHz frame rate. Figure 7 shows a schematic of the optical set-up (A) and a selected dataset (B).

It is important to note that this detection scheme operates under the grey body assumption, where the emissivity is assumed to be equivalent to the two wavelengths. Furthermore, the spatial resolution in this arrangement is on the order of 0.1 mm due to the detectors’ low pixel density. Higher spatial resolution may be achieved by using a detector with a higher pixel density, albeit at the expense of temporal resolution.

### 3.2. When the Grey Body Approximation Fails

**Figure 8:**
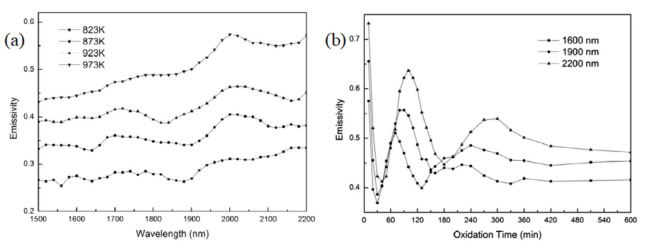
The emissivity of Ti6Al4V alloy as a function of wavelength/temperature (**a**) and oxidation time (**b**). Sample in (**b**) oxidized at 973K [70].

The two-color pyrometry method relies on the approximation that the material’s emissivity is constant at the selected wavelengths. The emissivity often has a strong wavelength, temperature, and compositional dependence, as seen in Figure 8.

**Figure 9:**
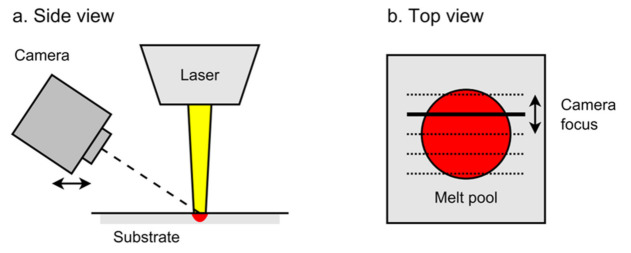
Schematic of hyperspectral pyrometric imaging [69].

In this case, the grey body approximation fails horrendously. Thus, two-color pyrometry is an inappropriate technique for accurate thermal characterization. Instead, multispectral or hyperspectral pyrometric imaging may account for the extreme irregularity in emissivity spectra. Devices et al. succeeded in approximating the local temperature of a melt pool of stainless steel using a hyperspectral line-scan camera (Figure 9) [69].

However, their analysis assumes a linearly decreasing emissivity spectrum appropriate for this material (liquid stainless steel) but is not highly generalizable to more complex alloys. It is possible to imagine that with careful calibration, a hyperspectral image data cube can be deconvolved via principal component analysis (PCA) and carefully collected calibration curves (with respect to temperature, wavelength, composition, and porosity, etc.). In this manner, approximations are rendered unnecessary, and an incredibly accurate determination of the temperature profile may be elucidated with spatial resolution. Hyperspectral imaging technology is still in its infancy and precludes the requirement for simultaneous spatial, spectral, and temporal resolution.

### 3.3. Discussion on Thermal Imaging

Commercially available thermal imaging cameras typically use a line-scan or push-broom technique wherein the camera focuses on a line. Refractive or diffractive optics disperse the resulting 1D image onto a 2D sensor. In this manner, the camera can collect one spatial dimension and one spectral dimension per frame. Generally, the line focus is raster-scanned across the object to collect a 2D temperature map. Due to the need for raster scanning, the spatial resolution of such thermal imagers is significantly reduced compared to modern imaging optics. A company called Photon etc. offers an alternative solution where a full 2D image is impingent on a holographic volumetric Bragg grating, reflecting only a single color (with an approximate spectral linewidth of 1 nm) onto a high-pixel-density detector. The volume Bragg grating is then swept across the desired spectral range. In this way, the company can collect thermal images with very high spatial and spectral resolution in a short time frame (<3 s for full spectral range). One of the main benefits of this design is the modularity in wavelength range, detector choice, and parameter settings.

Thermal imaging is one of the rare in-situ monitoring techniques applied in metal AM processing. The common purpose of thermal in-situ monitoring was to experimentally study the transient behaviors during the additive manufacturing processes, such as the melting pool shape in laser-melting AM and temperature distribution in solid-state AM. Although the spatial resolution of thermal imaging is not high, the instant or real-time imaging still provides irreplaceable information about transient behaviors, which can be barely obtained via other techniques. However, in the metal AM field, the limitation of thermal imaging is obvious: a lack of penetration. The thermal imaging systems can only capture the temperature distribution of the upper surface of the printing objects closer to the location of the thermal camera. In a thin fabricating sample, the obtained upper surface temperature distribution can be considered a representation of the entire depth. However, in a thick sample, the upper thermal imaging cannot represent the temperature distribution and the different depths under the upper surface. The metal material along the depth deviates from the temperature map from the upper thermal image to a very different distribution. The complex heat flux under the upper surface contributes to this. In a practical case, thermal imaging can monitor the horizontal melting pool shape on the upper surface. Nonetheless, it cannot estimate the melting pool depth information in laser-melting AM processes, which is a challenge in in-situ studies. The mentioned limitation of thermal imaging can barely be overcome due to the physical unfeasibility. Further improvement can be achieved using machine learning, which might pair the upper surface thermal imaging with the simulated temperature distribution of the entire object, which can be a potential study direction in the future.

## 4. Ultrasonic Inspection and Evaluation

Electromagnetic wave and mechanical tests are two primary inspection methods for AM-printed products. Electromagnetic wave uses X-rays or gamma-rays to provide information about the fraction of local porous volume. The mechanical test contains plasticity (hardness and strength) and elasticity (Shear modulus and Young’s modulus). Both methods are destructive either during testing or sample preparation, which leads to limited applications.

**Figure 10:**
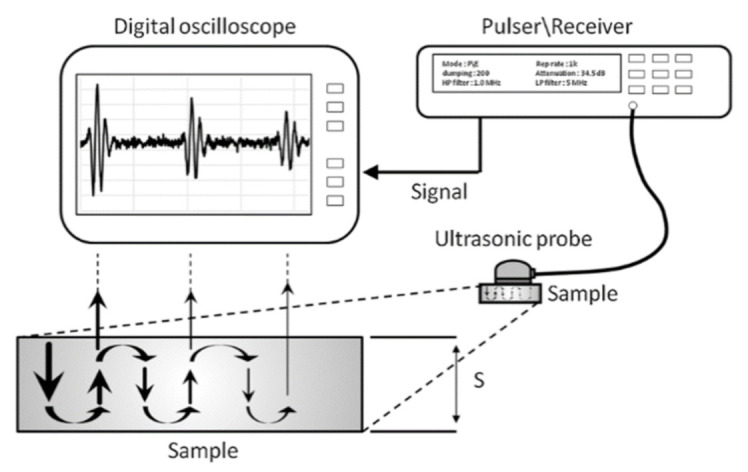
Typical ultrasonic non-destructive test set-up.

Ultrasonic testing, on the other hand, is widely used in non-destructive testing (NDT) for engineering applications [71,72] and medical diagnostics [73]. NDT techniques offer a cost-effective way of evaluating a sample for individual inspection or quality control systems of production. Ultrasonic NDT (Figure 10) includes identifying and characterizing materials to find defects in the interior, exterior, or both without altering or harming the sample.

Reflected waves (also called “pulse-echo” waves) and transmitted waves are two types of sound waves used to conduct ultrasonic testing. During ultrasonic testing, the wavelength of the ultrasound controlled by the frequency of the transducer is critical to detect discontinuities. If a discontinuity of the sample is greater than one wavelength, there is a reasonable chance of being observed.

Resolution is another crucial factor for the quality of ultrasonic testing. A common consideration for improving the ultrasound inspection resolution is increasing the sound wave’s operating frequency. However, a short wavelength of the sound wave introduces new inspection challenges, such as attenuation and dispersion of the ultrasound pulse, which introduces more uncertainties into the measurement. Specifically, the non-uniform microstructure could be comparable in the metal AM process as the high-frequency ultrasound wavelength. In such cases, attenuation and dispersion will negatively affect the resolution of ultrasound testing. Therefore, before performing ultrasound NDT evaluation, the basic knowledge of acoustics and metallurgy is required in order to ensure proper experiment design, including transducer, operating frequency, and coupling material selection.

### 4.1. Ultrasonic Diagnostics

A typical pulse-echo testing system has three components: pulser/receiver, transducer, and display screen. A pulser/receiver can drive the transducer to generate high-frequency ultrasonic energy by producing high-voltage electrical pulses. During testing, the acoustic energy travels through the sample as a broadband acoustic pulse envelope. Some of the power will be reflected if there is discontinuity within the sample. The size of detectable discontinuities should be at least 1.2 times larger than the operating wavelength of the sound pulse. A transducer can transform the reflected wave signal into an electrical signal, and the number, amplitude, and time delay data of the reflection signals will be displayed on a screen. Then, the calculation of discontinuity location, size, and other information is achievable.

The advantages of ultrasonic testing include:

– requiring a minimal part preparation procedure;
– not applying plastic deformation on the test material;
– good penetration depth;
– instant results.
– The disadvantages of the ultrasonic technique include:
– the surface must be accessible for ultrasound transmission;
– specific knowledge and training for operation;
– due to low sound transmission and weak signal–noise ratio, inspection at high frequencies is challenging due to the large grain size in cast raw material;
– standards of reference are needed for both calibration of equipment and defect characterization.

There are four main modes of sound waves in a solid, depending on how particles oscillate. Sound can travel as longitudinal waves, shear waves, surface waves, and Lamb waves in thin materials. Among these, longitudinal and shear waves are the two most commonly used propagation modes in ultrasound research.

Longitudinal waves are defined as waves that oscillate along the optical direction frontward and backward. Since these waves are involved in compression and expansion, longitudinal waves are also called tensile or compression waves. Sometimes, longitudinal waves are called pressure waves because the material density fluctuates with wave traveling. Longitudinal waves can cause propagation in fluids and solids because no shear motion is involved.

Transverse or shear waves oscillate through media at an inclined angle or cross in the direction of propagation. For efficient propagation, shear waves need an acoustically solid material and are not efficiently propagated in fluids and gasses without a shear modulus. As compared to longitudinal waves, shear waves are relatively weak. Moreover, shear waves are usually produced in materials that include some longitudinal motion energy.

**Figure 11:**
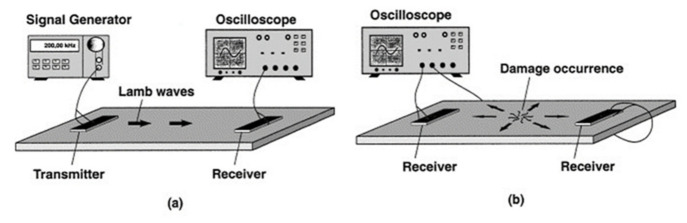
(**a**) Set-up of the Lamb wave analysis and (**b**) principle of the AE measurements.

Lamb waves and surface waves are commonly applied as combined modes of acoustic NDT test methods (Figure 11) [74,75]. The surface wave propagates on the surface of a medium, in which the transmission is highly dependent on the Young’s modulus of the medium. Lamb waves are dynamic vibrational waves spread throughout the material thickness parallel to the sample surface [76]. The difference is that lamb waves can only be generated in thin plates (a few wavelengths thick). The frequency of the test wave and the thickness of the material have a significant influence on Lamb waves.

**Figure 12:**
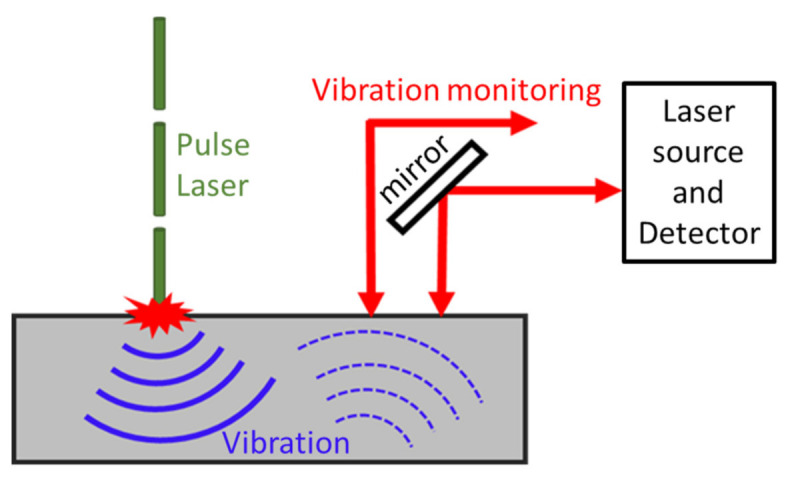
Schematic of laser ultrasonic set-up wave paths from top to bottom of a sample.

**Figure 13:**
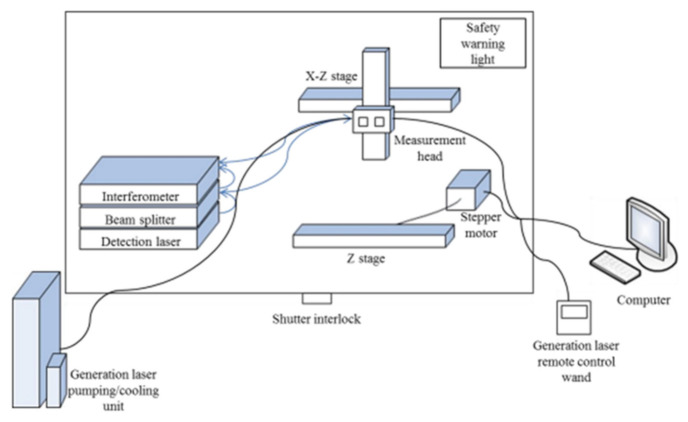
Schematic of laser ultrasound testing equipment set up.

Laser ultrasonic testing (LUT) is a non-contact inspection technique that demonstrates potential for evaluating the metallic AM process. LUT can be carried out on curved surfaces and in areas that are difficult to reach, even at high temperatures. For LUT, a laser pulse is focused on the sample surface, and an ultrasonic pulse is produced due to periodic rapid thermal expansion and contraction (Figure 12and Figure 13). The laser pulse produces Rayleigh or Lamb waves on a bulk sample with both transversal and longitudinal modes. The wave has scattering, attenuation, or diffraction when a defect has a size that is comparable to or larger than the operating wavelength. Defect information is produced to identify processing errors and modify processing parameters in real time [77]. The use of LUT generating a Rayleigh wave in power direct energy (PDE) deposition metallic samples has been done for a small number of studies in which discontinuities between 150 and 500 μm were observed at a depth of up to 700 μm, which were considered surface or close to surface defects [78]. Another work reported and proved the possibility of using LUT generating a Lamb wave to detect the natural formatted [79] and artificial [80] defects inside a printed Ti6Al4V block.

The size of discontinuities in the metal additive manufacturing processed products is highly dependent on the printing methods. The sizes of discontinuities generated during the selective laser melting process (SLM) and powder bed fusion (PBF) process are generally the smallest and most challenging for ultrasound-based non-destructive testing and diagnostics. The size of discontinuities is usually comparable to or smaller than the wavelength of the existing commercialized high-frequency transducers. There is barely any reported work on ultrasound NDT diagnostics on PBF and SLM products in the current literature. However, in other printing methods, such as wire arc additive manufacturing (WAAM) [81], very-high-power ultrasound AM [82], and friction stir AM (FSA) [83], ultrasound NDT testing and diagnostics could be suitable due to the larger products and size of discontinuities resulting from the techniques. A study reported that traditional A mode ultrasonic diagnostics in WAAM aluminum and steel products by 500 kHz transducer are successful [84]. The reflected waves occurred in discontinuities of a few mm wide and cm long [84]. For instant imaging inspection, a study reported the ultrasonic diagnostics of artificial cylindrical 3 mm defects designed and fabricated in a WAAM aluminum alloy by 10 and 12 element phased-array transducers, which worked at 3.25 MHz [85]. The echo intensity from the hollow defect was increased proportionally to the phased-array element numbers. The defect was clearly shown on the phased-array imaging with approximately the correct size.

**Figure 14:**
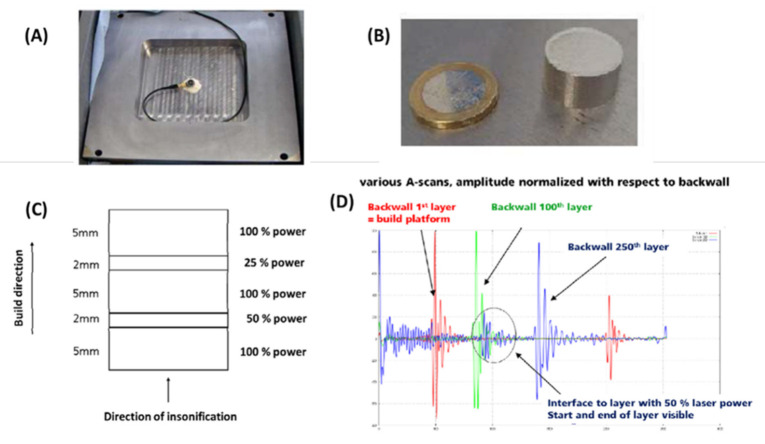
In-situ monitoring of the SLM printing process. (**A**) Attached ultrasound probe on the backside of the printing substrate. (**B**). The printed sample. (**C**) Printed object region demonstration. (**D**) The detected reflection signal from the interfaces between the layers in (**C**).

A single plane wave transducer can be used to perform in-situ monitoring by attaching it under the building substrate for printing simple object geometry. In Figure 14, recent work is shown that described the in-situ ultrasonic pulse-echo-based monitoring of an SLM printing disc sample [86]. The sample was fabricated as several regions along the building direction under different operating laser power percentages by varying the laser power at different layers. The interfaces between the regions provided clear reflections with a large acoustic impedance mismatch induced by the mechanical property difference [87]. The concept could be applied for future in-situ AM monitoring. However, the complex geometry would introduce difficulty in practical applications.

**Figure 15:**
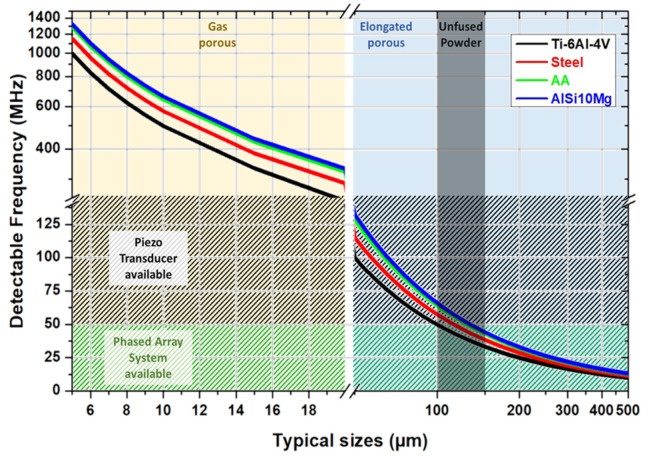
Theoretical detectable ultrasound frequency of gas porous (yellow shadowed), elongated porous (blue shadowed), and unfused powder (grey shadowed) in SLM-printed Ti6Al4V (black line), steel (red line), aluminum alloy (green line), and AlSi10Mg (bue line). 250 MHz and lower frequency range are available in the commercial piezo-element transducer market. 50 MHz and lower frequency range are available to obtain a commercial phased-array system.

In Figure 15, from an existing study [88], we show the range of typical discontinuity sizes in the powder bed fusion process and the speed of sound in common 3D-printed alloys, aluminum alloys, titanium alloys, and steel [12,89]. Based on this information, we can reverse the calculated equivalent operating frequency f table by using experimental literature speed of sound values c of the listed materials and equation c = f λ, where λ is the sound wave’s wavelength, representing the theoretical detectable minimum size of discontinuities.

**Figure 16:**
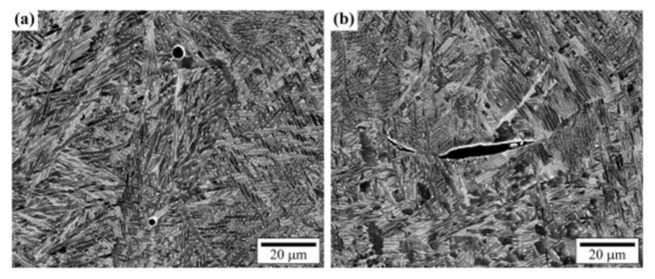
Examples of typical pores seen in SEBM deposits imaged by SEM (backscatter mode) in the x–z plane: (**a**) two circular pores and (**b**) a more irregular lack of fusion pore. The build direction is vertically upwards in the plane of the page.

A summary of commonly observed types and sizes of discontinuity in SLM-printed alloys compared with the alloys’ wavelength along the various frequencies is shown in Figure 15. In the plot, Ti6Al4V, steel, aluminum alloys, and AiSi10Mg were involved in the discussion. Due to the typical speed of sound in the alloys, the wavelength values of the sound wave were calculated from 5 to 1500 MHz. Based on existing studies, the typical discontinuity size range was summarized from 5 to 500 μm. Example pores are shown in Figure 16. From the theoretical calculation, the possible detectable frequency of the various types and sizes of discontinuities in the different alloys is plotted in Figure 14. However, due to the high speed of sound in the listed alloys, the detectable frequency of the small discontinuities was very high. Not all the frequency range is available in the commercialized ultrasound transducer market. The patterned area indicates the frequency range in which the commercial piezo-element transducers were available. The area patterned with the green lines refers to the frequency range available to obtain a phased-array system.

**Figure 17:**
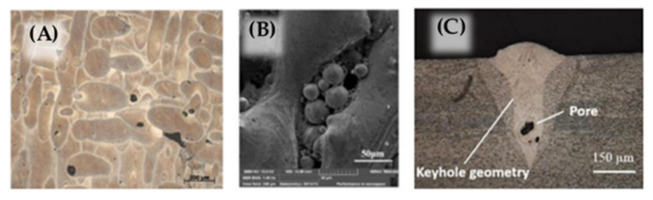
Spherical pores (**A**), unfused powder (**B**), and keyhole pore (**C**).

The unfused powder size in the printed products is usually 100 to 150 μm (Figure 17). The theoretical detectable operating frequency is from 30 MHz to 70 MHz, which can be easily detected by a high-frequency commercialized plane wave transducer such as 100 MHz or even higher. However, with a phased-array system, an instant 2D image non-destructive test (NDT) device, the current highest operating frequency is around 50 MHz [90], which is possible to find large-sized unfused powder around 120 μm to 150 μm in all listed common printing materials. In the case of small-sized unfused powder such as 100 μm, by the currently available highest-frequency phased-array system, the flaw can only be seen in the Ti6Al4V due to its lower speed of sound compared to other commonly printed materials.

Elongated pores caused by insufficient fusion between layers in the powder bed fusion process have a wide range of 50 μm to 500 μm [88] (Figure 17), which is also suitable for ultrasonic NDT tests. A 150 MHz fundamental frequency plane transducer can identify defects in all listed commonly printed alloys. Due to the limit of the available frequency range, if we want to use a phased array to test elongated pores, detecting the pores requires a greater resolution than the device could visualize at 275 μm without any advanced signal processing techniques.

As a commonly existing small discontinuity type during the printing processing [12,89], gas pores require ultra-high-frequency ultrasound to be detected, as the table illustrates. Due to the high sound velocity values in the commonly printed alloys, the theoretical detectable operating frequency is mainly above 250 MHz, which is currently the highest commercialized center frequency [91]. The 250 MHz fundamental frequency pulse could find larger gas pores only in Ti6Al4V and steel from the calculated value, which had a size around 20 μm. Any size of gas pores in aluminum alloys and small-sized gas pores in Ti6Al4V and steel are not possible to detect by ultrasound devices at the current stage. Since a 500 MHz or 1000 MHz ultrasound transducer is not commercially available, in other words, the small gas pores in printed products are not able to be detected by ultrasound devices.

**Figure 18:**
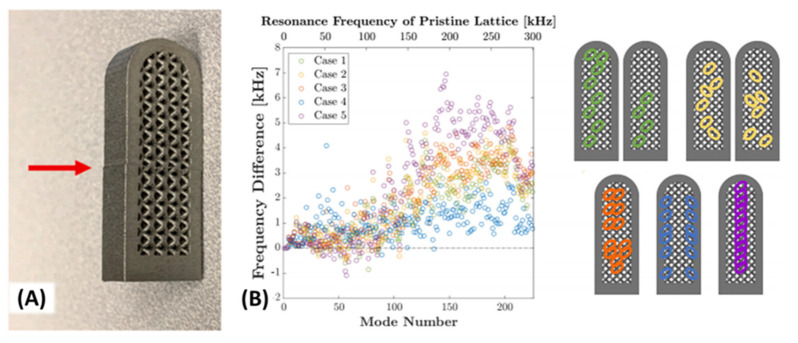
(**A**) Additive manufacturing printed sample with layer off-set flaw intentionally made by stoppage. (**B**) Numerically simulated eigenfrequency shifting of the object with different types and numbers of small intentionally introduced flaws.

As we summarized, the commonly observed defect size was smaller than the operating wavelength of the commercialized ultrasonic equipment, which limited the application of ultrasound NDT for flaw detection. However, besides the direct measurement determining that impedance mismatching occurred at a discontinuity, the acoustic wave can also be used in another way. In a macroscopy view, any solid objects fabricated by natural materials have vibrational resonance modes, which can be different due to the shapes and materials of the object. The unexpected flaws and defects will theoretically shift the vibrational resonance mode frequencies. For complex objects, the resonance modes are usually fewer and more discrete rather than a simple block. Coincidently, one of the original aims of developing additive manufacturing was fabricating customized and complex geometric samples. A reported study [92] described quality control inspection of metal alloy additive manufactured products with complex shapes (Figure 18). The numerical simulation estimated the resonance modes of the non-flawed sample. By comparing the frequency shifting between the numerically and experimentally obtained resonance modes of the sample, the flaws could be found with visible or invisible sizes. The methodology showed exceptional performance. However, from an inspection field view, the method can be a suitable technique to determine the flaw’s existence. To determine the specific type and locations of the flaws for industrial application, the sample preparation processes can modify the target and render the instantaneous inspection using ultrasound techniques redundant.

**Figure 19:**
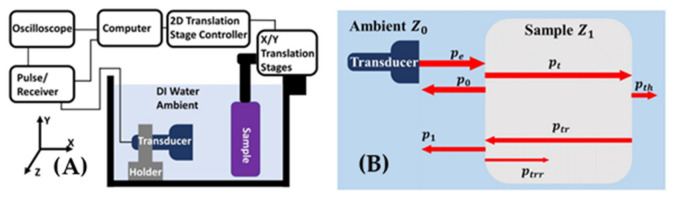
Effective bulk modulus elastography (EBME) technique. (**A**) Experimental set-up of the EBME. (**B**) The principle of EBME is determining the sample acoustic impedance and speed of sound to calculate the effective bulk modulus and effective density.

Nevertheless, besides the existing methods of flaw detection, a recently invented technique (Figure 19), effective bulk modulus and effective density elastography (EBME) [93,94,95,96], provided an alternative method for the detection of discontinuities in AM products by focusing on the local density fraction of the pores instead of seeking each pore. EBME can generate an effective bulk modulus (isotropic incompressible) and perform useful density mapping by measuring the sample’s acoustic impedance using longitudinal pulses in a monostatic set-up without any external stress. With the acoustic impedance of the ambient fluid material and sample thickness, the acoustic impedance of the sample material can be calculated. In the experimental procedures, with the impedance contrast between the sample material and reference ambient fluid material, the transducer-emitted pulse envelope separates in two echoes at the front and back interface between the sample material and ambient. With the time delay of the two reflected envelopes, the speed of sound in the sample material could also be determined. Once the acoustic impedance and sound velocity of the sample material were determined, the effective bulk modulus and effective density could be calculated by the equations: K = Zc and ρ = Z/c, where Z is the sample’s acoustic impedance, ρ is the effective density, and c is the sound velocity in the sample material. The method is limited to the use of ambient fluid, usually DI water. Only erosion-resistant material is suitable for use in the method, such as Ti6Al4V or most of the aluminum alloys. To satisfy the demand to test other alloys that are sensitive to water, such as steel or magnesium alloy, other ambient fluid materials with closer acoustic impedance to water could be involved in the inspection, such as alcohol. Instead of determining and localizing the individual small pores inside printed products, the EBME technique can provide a local density fraction. The local density fraction could inversely estimate the porosity by comparing the tested sample with a calibrated reference object in the same material. However, by the EBME technique, the ultrasound discontinuity detection’s wavelength/operating frequency limitation could be overcome by providing effective density information about the tested sample.

As a non-destructive acoustic test, EBME can generate a practical density map during the lateral axis scan, which shows the density gradient change for the ABS sample printed by the FDM method in both high-density contrast and low-density contrast experiments. The quality of 3D-printed objects can be examined by analyzing the effective density map since unexpected voids can change the local density of the sample. EBME may be used for ex-situ quality control in a non-destructive and less time-consuming way [97].

### 4.2. Non-Destructive Ultrasonic Elasticity Measurement and Elastography

The elastography techniques (M mode imaging) are usually applied in a soft material environment in the biomedical field. Due to the low Poisson ratio of industrial materials such as metal, alloys, and ceramics, the linear region elastic deformation on the materials is usually too small to detect by an external force. Hence, techniques such as strain mapping and Poisson’s ratio mapping, which require external stress and radiational stress, are barely applied to industrial materials or metal AM products. However, the shear-wave elasticity imaging techniques might be applied to the hard materials since the vibrational stress source generating small deformation is from another low-frequency ultrasonic transducer. The low-frequency vibrational deformation could be monitored by a high-frequency transverse wave in transient measurement. In metal AM products, the shear wave elasticity imaging or monitoring could be applied as an effective in-situ monitoring technique during the printing process. The evolution of a layer of metal prepared by the AM process can be predicted, and the high-frequency shear wave propagation within the metal might provide interesting details to predict the quality of the finished product. Based on the phase shift of the frequency components in the propagation of a shear wave, the heating effect on the deposited layers during printing might be determined using transient process techniques. Using shear-wave elasticity imaging leads to a minor impact on print processing due to the low-powered low-frequency vibrational stress. The absolute elasticity values from the SWEI might not be reliable enough to replace conventional mechanical testing techniques. The outstanding advantage of the SWEI in-situ monitoring is the transient elasticity behavior during the printing process. Once the technique is well-adjusted for metal AM, the elasticity transient behavior information could be invaluable for metal AM process parameter optimization. For determining reliable absolute values of elasticity for metal, alloy, and ceramics, so far, the existing elastography imaging technique is insufficient.

Besides the techniques adapted from the biomedical field, the industrial field has many existing ultrasonic non-destructive testing methods for examining the elasticity in ex-situ set-ups, such as longitudinal and transverse wave velocity techniques. With the known or assumed values of the sample thickness and sample density, the shear modulus and Young’s modulus values of the tested sample can be calculated by the time of flight of the longitudinal and transverse ultrasound waves.

### 4.3. Longitudinal and Transversal Sound Velocity Elasticity Measurement

Tensile tests and nano-indentation are two traditional destructive methods to obtain a material’s elasticity properties. Ultrasonic testing of longitudinal and transversal sound speed is the most common non-destructive method used to determine the material’s elasticity. Based on Hooke’s Law and Newton’s Second Law, the speed of sound in anisotropic media can be a factor linked to the elasticity and density of the media, which is expressed as $C={\left(E_{ij}/\rho \right)}^{-2}$, where C is the speed of sound in the media, ρ is the density of the media material, and $E_{ij}$ is the elasticity along with the wave oscillating direction.

In the conventional speed of sound elasticity measurement, density ρ and sample thickness d values needed to be pre-measured. By measuring the time of flight of longitudinal and transversal mode ultrasound in the two parallel surfaces of the sample in a monostatic set-up, the speed of longitudinal and transversal mode ultrasound can be obtained as $C_{L}=2d\;t_{L}^{-1}$ and $C_{T}=2d\;t_{T}^{-1}$, where $C_{L}$, $C_{T}$ are the speed of longitudinal and transversal mode ultrasound and $t_{L}$, $t_{T}$ are the time of flight of the sound wave in the sample. With the speed of transversal mode ultrasound $C_{T}$, the shear modulus G of the tested sample is calculated as $G=\rho C_{T}^{2}$. The Young’s modulus E and Poisson’s ratio ν of the measured sample can be computed by the known density and determining the shear modulus by $C_{L}={\left(\frac{E\left(1-\nu \right)}{\rho \left(1+\nu \right)\left(1-2\nu \right)}\right)}^{-2}$ and $G=\frac{E}{2\left(1+\nu \right)}$.

The method provides accurate elasticity when the tested sample is homogeneously compared to the ultrasound wavelength’s size, which is well applied in metals and alloys [98,99,100]. However, when the microstructure size approaches the wavelength of operation used for the ultrasonic characterization, the sample can no longer be approximated as a homogeneous medium. Due to the comparable size of the grain and the ultrasound wavelength, additional dispersion [93], attenuation [101,102], and scattering effects [103,104] impact the wave propagation in the results; the speed of sound in the media is wavelength-dependent. In this case, longitudinal and transversal mode ultrasound’s tested elasticity would be the dynamic modulus at the tested frequency. Moreover, when the microstructure of the tested sample is directional or anisotropic, such as a layer structure [105,106], the ultrasonic tested Young’s modulus and shear modulus are directional due to directional transversal mode sound velocity [107,108], which is obtained in ultrasound NDT in metal AM products [87].

**Figure 20:**
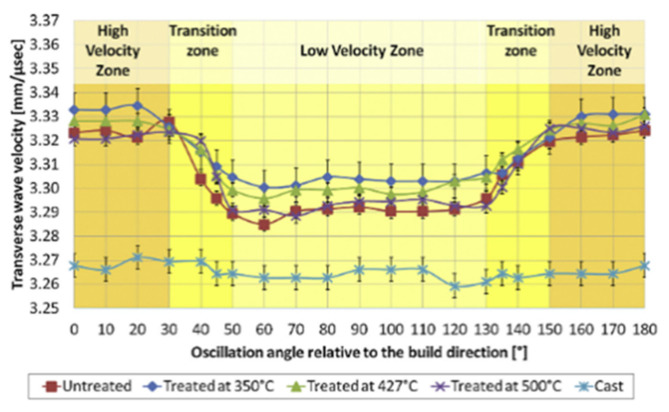
Orientation-dependent shear-wave speed in SLM-printed AlSi10Mg samples with and without additional heat treatments.

In a recently reported study, ultrasonic longitudinal and transversal mode speed of sound tests were performed on AlSi10Mg cubes printed via the selective laser melting process before and after heat treatments [87]. The values were compared with a reference sample from casting. With physically measured sample density and sample dimensions, the speed of sound values was tested by a 5 MHz shear wave transducer and a 20 MHz longitudinal transducer. The measured speed of longitudinal and transversal mode sound was directional (Figure 20); the longitudinal speed of sound was slower when the wave was propagating along the sample building direction. The transversal mode speed of sound was slower in the propagation direction, which is normal for printed layers. Moreover, both longitudinal and transversal mode sound yielded a greater velocity in the SLM-printed block than the casting reference block. From the heat-treated samples, the speed of the sound of both longitudinal and transversal waves decreased inversely proportional to the temperature of the heat treatments. For a known value of the density of the casting reference cube, which was larger than the density of the SLM-printed cubes, the study concluded that the elasticity of the SLM-printed AlSi10Mg cubes was greater than that of the casting AlSi10Mg cubes. These results were also in agreement with their ultrasound NDT experimental results.

**Figure 21:**
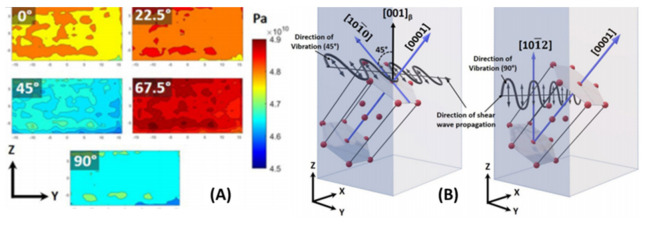
(**A**) Ultrasound scanned angle-dependent dynamic Young’s modulus distribution. (**B**) Hypothesis based on the experimental observation from ultrasonic techniques and conventional characterizations.

Besides the conventional ultrasonic testing methods such as the contact sound velocity measurement, the new technique presented other information related to the quality and even microstructure. A recent work [109] demonstrated the use of angle-dependent ultrasonic elastic modulus distribution to show the anisotropic response of crystal texture in an SLM-printed Ti6Al4V sample (Figure 21). The spatial map of the mechanical properties showed the homogeneity of the elastic modulus along the building plane and the building directions. The manufacturing stability of metal additive manufacturing products is important to study but proper and simple methods are lacking. The experiments in the literature demonstrate the feasibility of obtaining stable information.

Moreover, with the anisotropic shear modulus distribution, the observation showed strong angle dependence in the SLM Ti6Al4V products. On the side face along the lateral and building axes, the dynamic shear modulus values were lower along 45° and 90°.  This is explained by the experimental observation of the existence of a 45° anisotropic texture structure in the sample’s microstructure. The drop in overall dynamic elasticity was contributed by the scattering and attenuation effects from the sample microstructure. As observed, the 45° anisotropic texture structure was the source of a decrease in elasticity. In a bulk view of the sample, on the 45° wave polarization angle, a more significant effect was measured, which referred to a high possibility of the 45° texture structure. Additionally, the reduction in ultrasonic shear modulus along the 90° wave polarization angle matched with the softer direction of the $\alpha^{\prime }$ HCP crystal in the texture structure, which agreed with another observation [87] and common angle-dependent mechanical test results of the SLM Ti6Al4V products.

The principle of ultrasound wave transmission is atomic vibration propagation. Although larger-scale microstructural anisotropy leads to the presence of acoustic scattering and attenuation, the ultrasound propagation is directly proportional to the elastic constant of a medium, referred to as the atomic distance. This principle brought the ultrasonic nondestructive evaluation of elasticity to reality and led to its widespread use. Furthermore, the atomic distance variance in a certain type of metal or alloy can indicate residual stress. In the fusion-based manufacturing processes, residual stress can significantly affect the plasticity, such as strength, which can be understood as pre-loaded compression and tensile stress inside the materials. The local slight tightening and loss of atomic distance does not affect static elasticity but causes the high-frequency dynamic elastic constant to vary. Hence, further development in non-destructive ultrasonic residual stress evaluation could be a faster but less accurate alternative solution for the conventional neutron beam scan technique.

**Figure 22:**
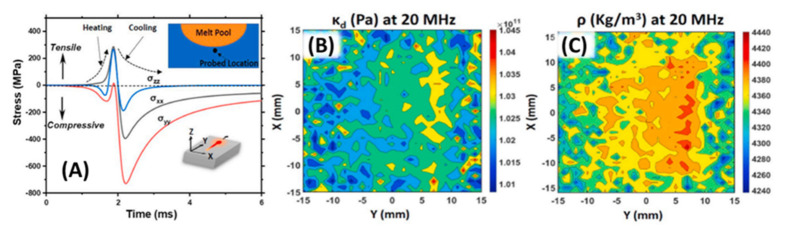
(**A**) Numerical computational results of residual stresses along laser scanning plane and building direction. (**B**) Dynamic bulk modulus elastography on the SLM Ti6Al4V sample along the laser scanning plane. (**C**) Effective density distribution on the SLM Ti6Al4V sample along the laser scanning plane.

In a recent work [110], high-frequency ultrasonic bulk modulus and effective density elastography techniques were applied to determine the thermomechanically induced residual stress from the SLM manufacturing process on a Ti6Al4V sample, which is illustrated in Figure 22. Based on numerical simulation, the work reported the compression and tensile residual stress from the rapid heating and cooling cycle along the horizontal and vertical directions. On the laser scanning plane, compressive residual stress was significantly higher compared to the vertical building direction. The numerical results were indirectly verified by ultrasound-determined elasticity and effective density maps. In the maps, the observation showed that the core part of the Ti6Al4V sample had a higher dynamic elastic constant and effective density than the part of material closer to the sample edges on the laser-scanned plane. Due to the forward–backward laser-scanned path on each building plane, the edges of the sample experienced U-turns on the laser-scanned path, decreasing the rate of heating and cooling, leading to slower melting/solidifying and lower compressive residual stress.

**Figure 23:**
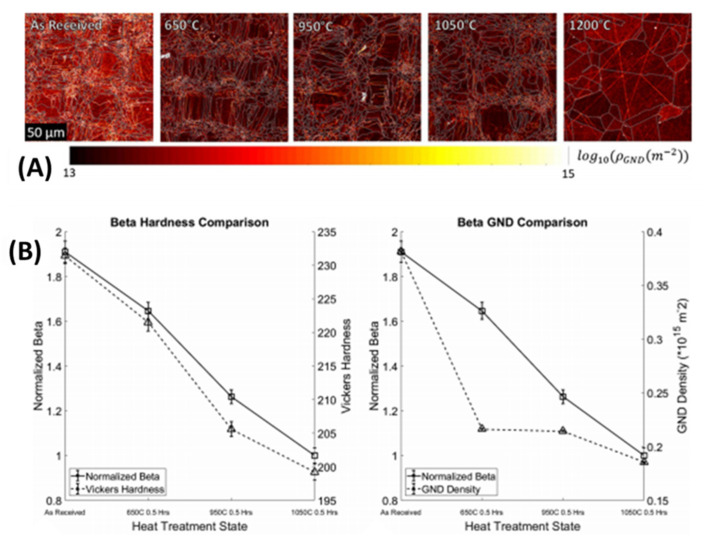
(**A**) The intentionally modified grain size of AM steel samples. (**B**) Experimental measurement of nonlinearity factor β versus different grain sizes from varying heat treatment states.

Besides predicting interatomic distance and crystal orientation using ultrasound, scholars have also demonstrated the use of ultrasonic measurement in the estimation of grain structure in AM-built steel samples [111]. The AM-built samples were intentionally heat-treated in this study to have different grain sizes (Figure 23). The microstructure size (plasticity: hardness) was estimated using surface-wave second-harmonic generation. It was observed to be inversely proportional to the nonlinearity factor β. The reported methodology perfectly measured the plasticity by ultrasound, which has never been demonstrated in any previous study. However, surface second-harmonic measurements can be challenging for actual quality control during any manufacturing process.

### 4.4. Comparison of Various Ultrasonic Techniques

Compared with electronic and optical inspection methods, the acoustic technique has inferior resolution. However, the speed of acoustic evaluation is outstanding. In metal additive manufacturing, conventional ultrasound diagnostic methods can be barely applied to small, printed objects from SLM and DED processing as the typical discontinuity size, such as gas pores, is much smaller than the wavelength of the high-frequency piezo-element transducer available on the market. The elongated porous and unfused powder is, in principle, detectable by transducers with a high fundamental frequency such as 125 MHz. Some larger sizes of typical elongated porous and unfused powder can be shown in instant imaging from a high-frequency phased-array system around 50 MHz. The EBME technique can yield useful information about small discontinuities from the effective density variations along the wave propagation direction instead of detecting small individual discontinuities. This might be feasible in the future, dependent on the developments in ultrasonic technology.

On the other hand, high-frequency ultrasonic diagnostic methods are considered suitable for quality monitoring and inspection for the relatively larger products from WAAM, FSAM, and UAM. Shear wave, lamb wave, and Rayleigh wave would be interesting to apply to the metal AM layer structure from different directions to determine the orientation-dependent elasticity difference between the horizontal, vertical, or even some specific angles.

Ultrasonic non-destructive tests to estimate mechanical characteristics are commonly applied in industries. In metal additive manufacturing, these techniques are barely used since the current stage of metal additive manufacturing is still primitive from the perspective of material diagnostics. Printing a tensile test sample is convenient for obtaining results such as Young’s modulus and Poisson’s ratio (Elasticity), strength, and failure elongation (plasticity). The ultrasonic test does not provide any information on plasticity since it is non-destructive. The techniques are more suitable to apply for products that are needed for further use. However, once metal additive manufacturing develops towards the common use of FDM plastic printers, non-destructive elasticity evaluation would be necessary to apply to the products before their actual use.

Elastography imaging is a category of commonly used elasticity measurement techniques in the biomedical field. The methods are usually applied in a soft and organic material environment. Since some of the methods require external stress to provide detectable ultrasound deformation, such as the strain maps, the methods cannot be applied to metal AM products due to the small accessible linear elastic strain in metals and alloys. Nevertheless, the shear-wave map techniques might be used on metal AM products since the principle of the techniques uses a high-frequency shear-wave to monitor the sample vibration from a low-frequency source. As far as using medical imaging devices on metals, the conventional medical probe coupling gel could not be applied since its close-to-water acoustic impedance is highly mismatched with metals and alloys. Honey or other viscous liquids with high acoustic impedance values could be used. In the future, effective bulk modulus elastography could be used to determine the dynamic bulk modulus. Combining the EBME and shear-wave map, the collected bulk modulus and shear modulus can be used to calculate other elasticities such as Poisson’s ratio and Young’s modulus. Ultrasonic longitudinal and transversal mode speed of sound tests have already been used on SLM-printed products, showing outstanding results on the absolute values of the elastic modulus with the known size and density of the samples. The orientational shear-wave velocity showed stronger elasticity in the horizontal direction and weaker elasticity in the vertical direction, which means that it could be a promising alternative method to the tensile tests on directional printed samples.

Ultrasonic inspection is the most commonly applied non-destructive test technique. Unlike most other categories of inspection methods, such as mechanical or electromagnetic wave-based inspection, the resolution of ultrasound tests is closely related to the sample conditions, including the material composition, sample surface, sample thickness, and sample geometry. In different materials, ultrasound has significantly different speeds of sound and a frequency-dependent dispersion effect, which dramatically changes the operating wavelength of the ultrasound test with a certain frequency. The common resolution of the ultrasound test is close to the operating wavelength. Hence, without a complete understanding of the speed of sound and the frequency-dependent dispersion effect [112] in the tested materials, the ultrasound test might involve non-negligible uncertainties in the test resolution, including the flaw and elasticity. The limitation might be overcome by using a known sample as a reference or performing a thorough characterization of the acoustic properties of the involved materials. In addition, the surface roughness and geometric complexity can also vary the ultrasonic evaluated internal discontinuity and elasticities with the contacting experimental configurations. An immersion test could be a potential solution to overcome this challenge. To increase the inspection resolution, recently developed acoustic metamaterial lenses [113,114,115,116] can offer sub-wavelength resolution in ultrasound inspection.

## 5. Mechanical Test

Mechanical testing plays an important role in the diagnostics of AM products. Most of the mechanical tests have been applied for decades and are standardized in industrial fields. We have summarized and compared some typical mechanically tested elasticities and plasticities from the literature on some AM products in plots. As the conventional ex-situ quality monitoring methods, the mechanical tests can be categorized into the following types.

### 5.1. Elasticity Test with Information on Plasticity

A tensile test is a fundamental engineering and material science test in which a sample experiences a controlled uniaxial load until failure. After performing the tensile testing, a stress–strain curve will be generated. The mechanical properties, such as ultimate tensile strength (UTS) and maximum elongation, can be directly measured through a tensile test. The tensile test can be used to derive some other properties, such as Young’s modulus, Poisson’s ratio, yield strength, and strain-hardening characteristics [117].

**Figure 24:**
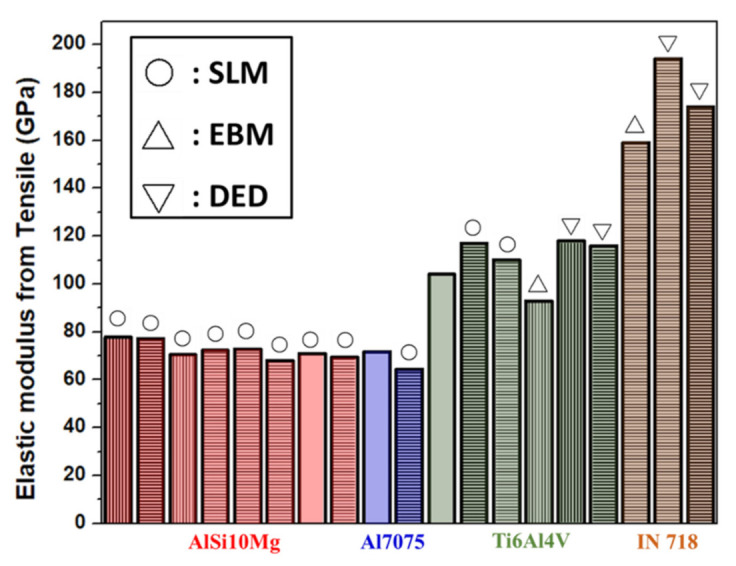
Selected values of tensile elastic modulus from existing literature on metal AM. The horizontally patterned bar indicates horizontally printed samples, and the vertically patterned bar indicates vertically printed samples Cast reference values are represented without a pattern [83,118,119,120,121,122,123,124,125,126,127,128].

Figure 24 shows all the cited tensile elastic modulus values of AM metals from the existing literature. In aluminum-based alloys, the variation in the tensile elastic modulus was within 8%. Regarding results of testing Ti6Al4V, the variation reached approximately 15%. The differences between the values were related to the printing method. SLM and DED fabricated products with a higher elastic modulus than other methods. From the values for DED-printed Ti6Al4V, the value for the vertically printed sample was higher than that of the horizontally printed sample, which was a surprising result.

Moreover, in IN 718, a tensile elastic modulus of more than 18% was found among the literature values. It is difficult to draw conclusions about the more considerable variation in the tensile-tested elastic modulus of Ti6Al4V and IN 718. Some of the tensile-tested elastic modulus values within the literature were due to different processing procedures or experimental uncertainties. However, due to the high elastic region stress–strain ratio, the brittle alloys are less suitable for elasticity calculation by tensile tests. An extensive increase in occurs in a limited elongation, which requires a highly accurate loading cell and motion driver in the mechanical testing machine. AlSi10Mg, Al7075, Ti6Al4V, and IN 718 are closer to brittle alloys than steel or bronze; hence, the tested tensile elasticity might introduce more uncertainty into the modulus results.

In contrast to the tensile test, a compression test evaluates a material’s capability under compression load. During the compression test, the material experiences opposing forces that push the specimen inwards. The test sample is typically positioned between two plates that spread the applied load over the entire surface area and then move them through a mechanical testing system to flatten the sample. A compressed sample is typically shortened in the direction of the forces applied and extends perpendicular to the force.

**Figure 25:**
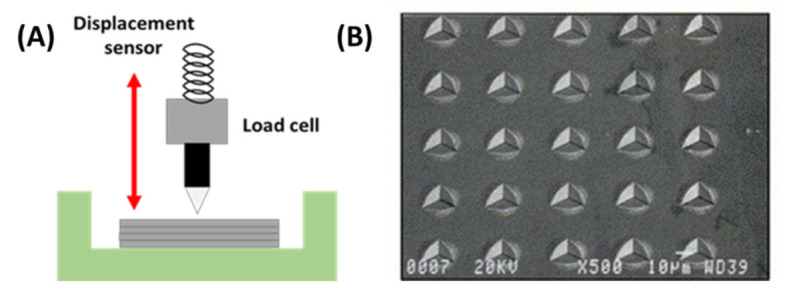
(**A**) Schematic illustration of an instrumented indentation system. (**B**) Indentations are a distance of 10 μm apart on a square of approximately 40 × 40 μm.

The idea of nanoindentation came from an indentation test and is an excellent way to test the mechanical properties, such as the Young’s modulus and hardness, of the thin films and surface layers of different materials, as shown in Figure 25A [129,130]. The nanoindentation is a test in which the measured penetration length is not millimeters but rather nanometers or micrometers. This test can be used to measure the mechanical properties of a smaller sample [131]. The critical item to classify the material properties is calculating the contact area for nanoindentation [130]. The typical tool head size is around the μm scale, as shown in Figure 25B. In nanoindentation studies, pyramidal indenters are used to achieve plasticity at shallow penetration depths and have been used to test thin films’ mechanical properties [132].

The SLM process can reliably achieve high dimensional precision and geometrically complex components without post-processing, which traditional methods cannot accomplish [133]. The repeated rapid heating and cooling cycles would also lead to residual thermal stresses in the components, which significantly affect the finishing and geometric resolution of the components. During various thermal or mechanical processes, residual stresses may be incorporated into mechanical components; such processes include heat treatment, shaping, and welding [134,135].

Assessment of residual stresses is important in additive manufacturing because it can positively or negatively affect the reliability and lifespan of components and devices. As a result, various techniques have been developed for measuring residual stress, such as X-ray and neutron diffraction, ultrasound speed, penetration, and layer removal techniques [136].

In the study [137], residual stresses were found to be due to repeated thermal cycles from the manufacturing of electron beam additives (EBAM) and the selective laser melting process (SLM). The EBAM and SLM parts Ti6Al4V and Inconel 718 were studied for their residual stresses using a methodology developed by Carlsson et al., a mechanically instrumented indentation technique based on the experimental correlation between indentation property and residual stress. The result showed that the compressive residual stress of the Ti6Al4V EBAM components was present in both the Z-plane and X-plane and that the Inconel 718 SLM components were tensile and compressive in the Z-plane and X-plane, respectively. In comparison, the Ti6Al4V components had lower total residual stress than the Inconel 718 components. In addition, the Vickers hardness values in the parts developed with SLM and EBAM were comparable to the literature results.

**Figure 26:**
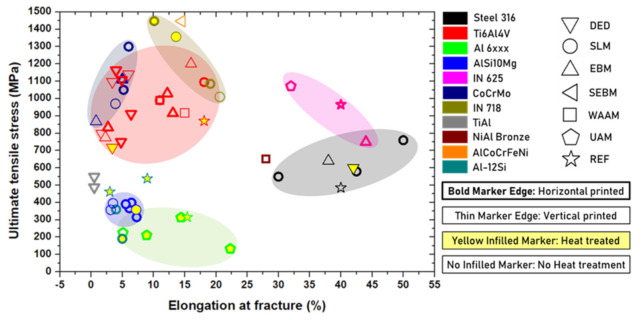
Selected values of fracture elongation verse ultimate tensile stress of various alloys from existing literature on metal AM [118,119,120,121,122,123,124,125,126,138,139,140,141,142,143,144,145,146,147,148,149,150,151,152,153,154,155,156,157,158,159,160,161,162].

Besides the effects of discontinuity in the tested AM alloy sample, there are unique testing methods to distinguish whether a material sample is brittle or ductile. Figure 26 shows the tensile-tested ultimate tensile stress verse elongation at fracture of various additive manufactured alloys from the existing literature. Moreover, Figure 26 summarizes the testing results from the existing literature on the brittleness of the alloys, which not only contributed to the material compositions but also the manufacturing processing procedures. The figure shows that for AlSi10Mg, horizontally printed samples had larger elongations at failure than vertically printed samples under the same printing method. In IN 625, highlighted in pink, the plot shows that UAM led to greater brittleness than the casting reference as a solid-state AM method.

Another useful piece of information could be exacted for the field of additive manufacturing. In the Ti6Al4V summary, DED fabrication yielded brittle samples. For the melting AM technique, EBM offered a more ductile IN 625, which showed larger elongation at failure. EBM and SLM yielded larger failure elongation values that approached those of the casted reference Ti6Al4V specimen.

**Figure 27:**
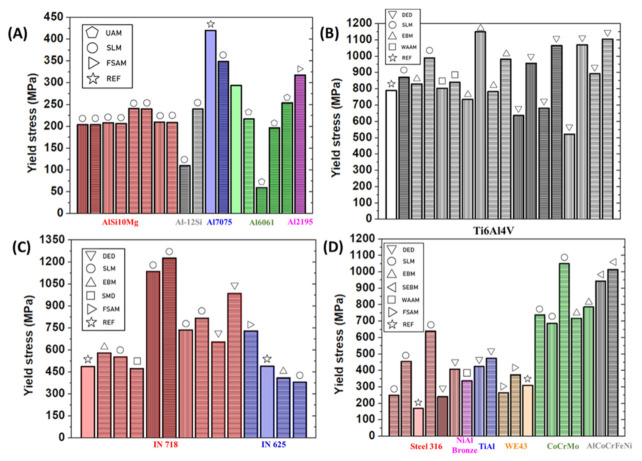
(**A**) Selected values of the yield stress of aluminum-based alloys from existing literature on metal AM. The horizontally patterned bar indicates horizontally printed samples, and the vertically patterned bar indicates vertically printed samples. [118,119,120,121,152,160,161,162,163] (**B**) Selected values of the yield stress of Ti6Al4V alloys from existing literature on metal AM [122,123,124,125,126,127,140,141,142,143,144,145]. (**C**) Selected values of the yield stress of nickel-based alloys from existing literature on metal AM [83,128,139,147,156,157,158,159,164]. (**D**) Selected values of the yield stress of other alloys besides aluminum-based, nickel-based, or Ti6Al4V alloys from existing literature on metal AM [138,146,148,149,150,151,152,153,154,165,166,167,168]. The bolded pattern indicates a heat-treated sample.

Yield strength indicates the maximum allowed stress in the linear elastic region, usually determined from tensile testing. Figure 27 includes various alloys’ tensile-tested yield stress, a transient point between elasticity and plasticity. The typical experimental approach to examine the yield strength finds the point where the stress vs. strain slope changes. 

Similar to testing the elastic modulus, the yield strength point occurs around the end of the linear stress–strain region. The tensile test machine’s high-precision loading cell and motion motor are required to accurately determine the starting point of the slope change on the tested stress–strain result line. The leak of optimization on sample and loading cell selection might introduce uncertainties to the measurements. In the aluminum-based alloys, SLM-manufactured AlSi10Mg samples showed stable yield stress values. UAM-printed Al6061 had considerable yield stress variation, which might be due to the existence of discontinuities. In Ti6Al4V, DED and EBM led to a larger variation in the tested yield stress values than the SLM technique. IN 718, as a high-elastic-modulus material, had almost the highest yield stress values in AM-produced specimens than the casted reference. The DED-manufactured samples showed more than 25% yield stress difference between horizontally and vertically printed samples. Besides the discontinuity effects, the partial difference might be introduced by the uncertainty from the loading cell or motion motors. The results for the more ductile material IN 625 suggest higher yield stress in the FSAM sample manufactured using solid-state AM techniques and lower for the powder melting-based technique compared with standard reference sample values for conventional techniques. Similar to IN718, CoCrMo alloy, another brittle alloy, showed more than 25% yield stress difference between horizontally printed samples using the same technique.

**Figure 28:**
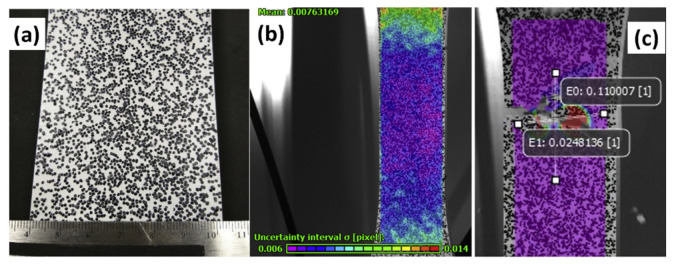
(**a**) Speckle pattern applied on the tensile test specimen. (**b**) Uncertainty in the correlation of subsets in images. (**c**) Virtual extensometers were placed on the fracture site [169].

The digital imaging correlation (DIC) tensile test is an optical technique that uses digital cameras to track visible features on a specimen to generate full-field strain and displacement maps. This technique enables researchers to analyze many advanced strain characteristics. When performing DIC, a specimen must be marked with a random pattern of contrasting marks with ink or paint. The test frame must be equipped with an advanced camera to record images for correlation. The images are divided into multiple subsets, each containing a pattern of features with a corresponding reference image. The tracking of features is accomplished by measuring the change in grayscale levels within each subset at every pixel’s n-number. Figure 28 shows the sample preparation and recording process of DIC.

**Figure 29:**
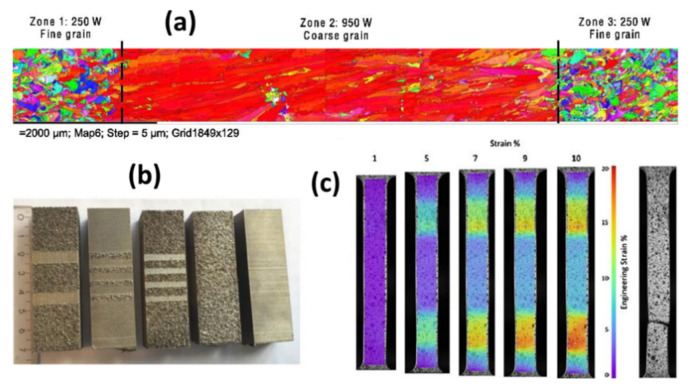
(**a**) EBSD analysis of a graded Inconel 718 specimen with a single coarse columnar grained zone embedded in a fine-grained matrix. (**b**) Pictures of different gradients in processed samples, depicting areas of 950 W (dark areas) and 250 W (bright areas). (**c**) Strain evolution as a function of crosshead displacement for the gradient with fine-grained matrix in the middle and coarse-grained regions on two sides [172].

During the additive manufacturing process, printing parameters and laser power and speed will affect the components’ texture, which influences the parts’ mechanical properties. Although the SLM parts have exceptional mechanical properties [170], they also have significant anisotropy owing to directional columnar grain formation induced by directional thermal conduction during the SLM process [171], which has a substantial influence on the mechanical properties [172]. Figure 29 shows the result of the DIC of the strain evolution in the gauge length of tensile samples as a function of applied crosshead displacement. The plastic deformation distribution is clearly shown, which was contributed by the significantly different sizes of grains tuned by laser power. The color distribution is also helpful for predicting the crack initialization located at the mismatched elasticity region [173].

### 5.2. Mechanical Test Provides Only Plasticity

Hardness is another important mechanical property that implies resistance to localized plastic deformation in a material. Brinell, Vickers, Knoop, and Rockwell are four types of hardness tests. The hardness value is generally defined as the indentation load ratio and the residual indent surface or projected area [174]. 

The technique of static indentation hardness is a standardized method for measuring the strength and fracture properties of brittle materials. In metals testing, indentation hardness is as common as tensile strength [175], although it is an inconclusive correlation and often restricted by geometry in terms of ranges of strength and hardness. In advances, the technique can also be used in raster-maps to provide the hardness distribution in a horizontal plane. The hardness distribution contrast in the products can provide valuable information on the manufacturing processes and the micro-structure deviation behaviors. Unlike tensile tests usually providing bulk-scale plasticity, frequently used micro-hardness offers local plasticity, corresponding to more information about the microstructure and dislocations. 

**Figure 30:**
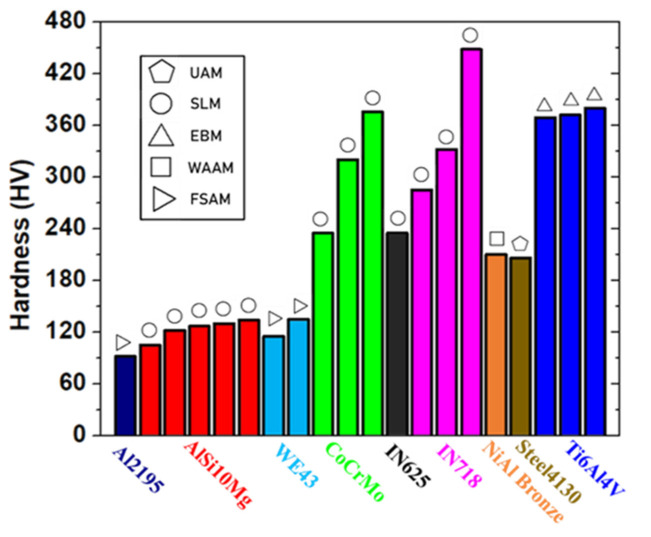
Selected values of hardness values from existing literature on metal AM [119,120,125,141,142,147,149,150,159,162,163,167,168].

Figure 30 shows the summarized literature hardness values of the AM products. This figure shows that AlSi10Mg and Ti6Al4V have stable tested hardness values, with CoCrMo and IN718 values vary considerably. This indicates that the plasticity of CoCrMo and IN718 alloys is much more dependent on the processing procedure. In the literature cases, a small difference between the laser power or scanning speed in the SLM process could offer significantly dissimilar plasticity in CoCrMo and IN718.

**Figure 31:**
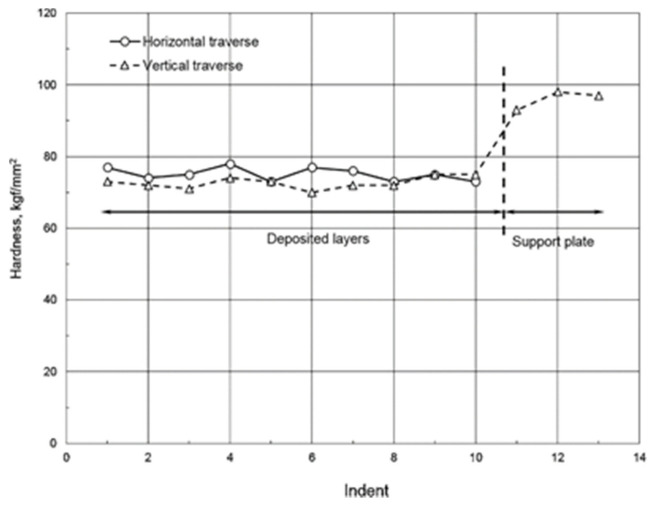
Horizontal and vertical hardness traverse perpendicular to the deposited layers.

Horgar et al. [176] explored the wire arc additive manufacturing of aluminum alloy AA5183 with traditional gas metal arc welding on a 20 mm AA6082-T6 platform. The hardness measurements in the horizontal and vertical cross-section of the flange sample are presented in Figure 31.

### 5.3. Impact Test

**Figure 32:**
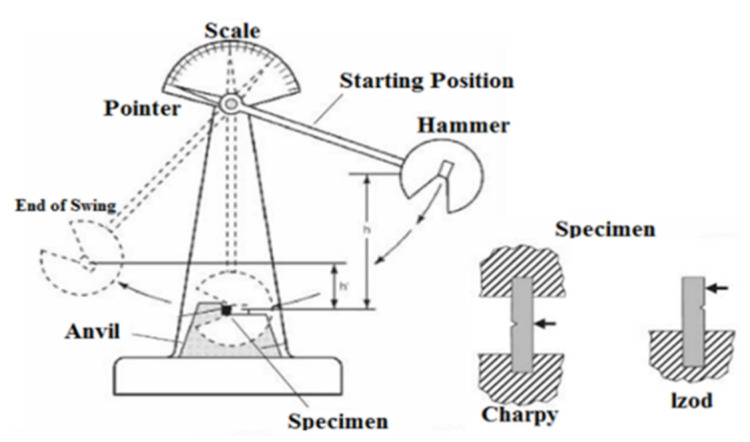
Charpy impact testing and Izod impact testing arrangement and sample design.

Impact tests (Figure 32) were established to identify the characteristics of materials’ fracture under high loading rates, deformation at low temperature, and triaxial stress conditions. Charpy and the Izod are two standardized tests to measure the impact energy.

The Charpy impact testing (CIT), also known as the Charpy V-notch test, has been used for the hardness assessment of metallic materials for over one hundred years [177,178]. The simple, fast, and economical testing method makes it standardized and widely used in the industry. CIT is a destructive test using specimens with a standard dimension of 10 mm × 10 mm × 55 mm according to ASTM A370 [179]. CIT determines the amount of energy absorbed by a standard notched specimen during breaking under an impact load. CIT also can help to establish the transition temperature for a material between brittle and ductile failures. CIT is primarily used in carbon steels and low-alloy steels [180].

**Figure 33:**
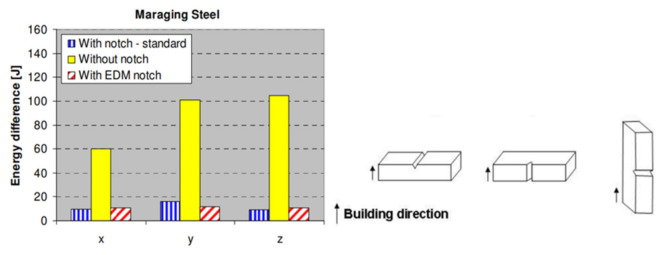
Amount of energy absorption measured by Charpy test on AM steel.

While using the Charpy impact test in the AM industry, an interesting study investigated energy absorption on directional printed AM steel samples, as shown in Figure 33. The test results showed lower energy absorption when the impact direction was normal to the building direction and higher energy absorption when the impact direction was normal to the built planes without a notch on the sample [181]. The energy absorption was higher when the notch was along the building direction and lower when the notch was along the built planes.

The results of the impact tests are more qualitative and not very helpful for design. However, one important function of the Charpy and Izod impacts tests is to determine, with decreasing temperature, whether a material experiences a ductile-to-brittle transition and the range of the temperature in which it occurs.

### 5.4. Fatigue Test

**Figure 34:**
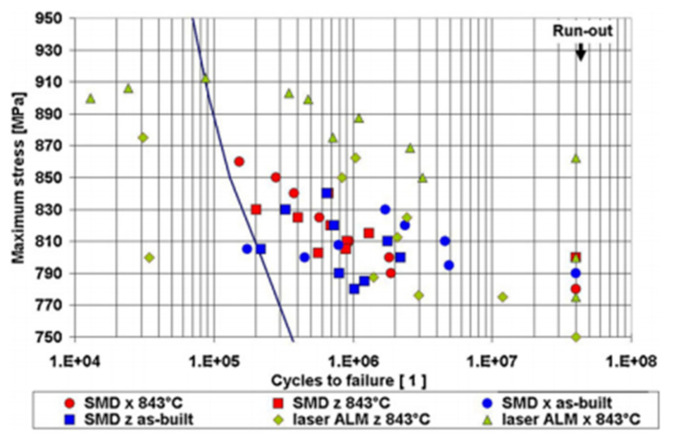
High cycle fatigue properties of a laser beam and SMD ALM specimens, tested along (x) and across (z) deposition direction; one data point represents one tested specimen. The line represents the upper fatigue limit required for wrought annealed material.

Fatigue is an important failure type that occurs in structures under dynamic and fluctuating stresses. Fatigue can be significantly lower than the yield or tensile strength under static load conditions at a stress level. Thus, fatigue is the largest cause of failure in metal, estimated at over 90% of failures [182]. Fatigue tests are performed to evaluate the stiffness and strength loss of materials under a continuous load and determine the cumulative number of load cycles in case of failure [183]. Repeated stress, compression, strain–compression, or other cyclic loading combinations are used to conduct fatigue tests. Repeated loading can damage the materials, which results in a loss in strength and eventually leads to complete failure. In some areas, materials must withstand repeated loading for an extended period, such as in the aerospace industry: a modern jet engine and airframe need to work somewhere from 15,000 to 20,000 h and 80,000 to 120,000 h [184], likes the Figure 34 illustrated. Therefore, a fatigue test is vital for these industry areas.

The fatigue resistance of materials is determined by the fatigue life curve (S–N). The curve is a plot with the average stress S relative to the number of load-to-failure cycles of material N. The curve is usually defined as a linear scale for fatigue stress and a log scale for load-to-failure cycles [185].

A study was conducted to evaluate defects’ influence on the axial fatigue strength of maraging steel samples manufactured by SLM [186]. Specimens with the longitudinal axis perpendicular and parallel to the building direction were examined. At the beginning of the tests, the microstructure of the materials remained in the as-manufactured condition. As a result, both orientations showed similar fatigue strength at approximately 30,000 cycles of fatigue life. However, the fatigue strength in the parallel direction became increasingly higher at longer fatigue lives. Moreover, the fatigue strength of the same material in annealed conditions was higher than the AM product. Another study of AM Ti6Al4V fatigue behavior compared the powder deposition technique and the laser melting technique [187]. The results in Figure 34 show that laser melted Ti6Al4V achieved higher accessible stress in fatigue tests, with more considerable variation between the products, than powder deposit processing. The horizontally printed sample allowed higher working stress than the vertically built sample, with less decreasing behavior in failure cycles and increasing the maximum stress. In the powder-deposited built samples, the heat treatment could increase the performance of some samples to have a larger maximum working stress. The investigated fatigue behavior of AM alloys has barely been studied in the existing literature; however, it is important and necessary to characterize it in order to improve its reliability and ensure that it can be applied in practical industries.

### 5.5. Advantages and Disadvantages, Comparison and Discussion of Mechanical Testing

As the most commonly applied ex-situ characterization and inspection techniques, mechanical testing methods provide unique information by different processing methods. In the category of elasticity testing methods, the tensile test, compression test, bending test, and nano-indentation test are usually used. Tensile, compression, and nano-indentation tests provide Young’s modulus and bending test results in a flexural modulus, which is considered another representation indicating similar information as the Young’s modulus in metals and alloys. The tensile, compression, and bending tests examine bulk scale elasticity; micro-tensile and nano-indentation tests analyze the smaller elasticity scale. In this sense, bulk-scale tensile, compression, and bending tests are more suitable to characterize AM products and materials and perform parameter-dependent studies. Moreover, the small-scale micro-tensile and nano-indentation are preferable to be used to determine the fundamental aspects in AM processing, such as phase transaction, grain sizes, and residual stress behaviors. The plasticity of metals and alloys is essential to characterize the allowable stress on the object without critical irreversible deformation or elongation. In the metal AM field, the plasticity of the printed objects is even more important to study compared with the conventional techniques developed to examine the limitations of the current stage AM techniques. Tensile, bending, and indentation tests are frequently applied to obtain plasticity information such as yield stress, ultimate stress, and hardness.

As mentioned in the previous paragraphs, the accuracy of mechanical tests could be affected by the equipment selection, especially in tensile tests. The equipment selection needs to be optimized in terms of its loading cells and the accuracy of the servo motors. For testing brittle alloys, optimization would be more critical to obtain accurate elasticity and yield stress results due to the sharp slope of the stress vs. strain curves. In addition, the tensile test is still a convenient method to examine several properties in one test, such as modulus, yield stress, and ultimate stress. The AM samples of the tensile test are usually prepared by direct printing. The samples’ preparation is less complicated for the tensile test than nano-indentation samples, which require cutting and polishing.

The test methods listed in the last paragraph are all static or involve low-strain-rate mechanical testing. For higher strain rates, such as fatigue and impact tests, the material could behave differently than in the static situation. The typical metals and alloys produced by conventional techniques do not significantly differ in response to low and high strain rates compared to viscoelastic materials such as rubbers and gel. However, in metal AM, inhomogeneities could be introduced in the products due to unsuitable printing parameters, unstable equipment operation, and flaw-based materials. The life cycle number, location, size of internal cracks, and the energy absorption from high-strain-rate dynamic testing are significant for AM alloy and metal products. The high-strain-rate fatigue test results could correlate to the type and size of discontinuities and inhomogeneities.

## 6. Laser-Induced Breakdown Spectroscopy

Laser-induced breakdown spectroscopy (LIBS) is an analytical technique that allows for the quantitative determination of atomic species in the analyte. In short, a high-power laser beam is used to ablate the material at the focal spot. The interaction of the ablated material and the surrounding atmosphere generates a high-temperature plume of plasma. Upon relaxation, the electrons, ions, and excited species in the plasma plume emit light via spontaneous emission, identifying the constituent atomic and molecular species [188]. The physical mechanism for LIBS is exceedingly complex and hence will not be discussed herein. Instead, we refer the reader to two comprehensive reviews by Hahn and Omenetto. The first outlines some of the basic physics within the plasma–particle interaction and diagnostics [189], while the second summarizes instrumental and methodological approaches to material analysis [190]. The experimental parameters for quantitative micro-analysis via LIBS vary greatly depending on the material being investigated. Parameters such as laser wavelength, fluence, pulse duration, observation interval, experimental geometry, and ambient environment must be optimized for a specific investigation. The review by Tongnini et al. provides an overview of these experimental approaches [191].

LIBS is an effective tool for the real-time characterization of additive manufacturing processes. Lednev et al. have demonstrated the feasibility of in-situ LIBS quantitative elemental analysis in a coaxial laser cladding technique [192]. Furthermore, the authors demonstrate that in-situ LIBS can be used for real-time process failure detection. In addition, Shin et al. have demonstrated composition monitoring using plasma diagnostics during a direct metal deposition process [193]. Although the spectral resolution in this study is far lower than standard LIBS, the authors can determine the ratio between Ni and Cr emission intensities as a function of composition, allowing for the in-situ determination of composition.

**Figure 35:**
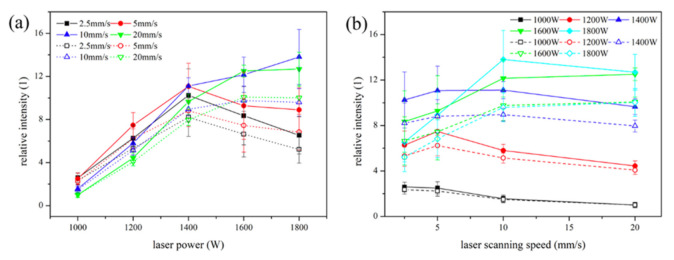
The correlation between relative mean intensity of spectral lines and laser parameters (solid points: Cr I, empty points: Fe I), (**a**) relative mean intensity and laser power, (**b**) relative mean intensity and laser scanning speed [194].

Recently, Wang et al. have monitored the plasma emission of AISI4140 steel during real-time laser processing, as shown in Figure 35 [194]. In this work, the authors monitored the micro-hardness of the molten zone as a function of various laser parameters. Using dimensionless analysis, the authors correlated the intensity of plasma spectral lines to predict micro-hardness with a mean prediction error of less than 3.1%. The results of this study are presented in Figure 35.

**Figure 36:**
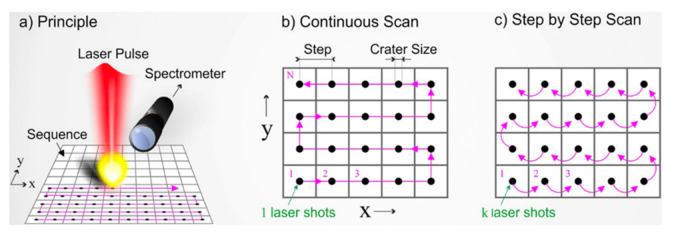
Principle of LIBS scanning and principal configurations of the measurement [195]. (**a**) Raster scan in the laser based AM process. (**b**) Continuous scan path. (**c**) Step-by-step scan path which was not continuous.

LIBS is inherently a single-point measurement, but advances in the speed and sensitivity of commercially available detectors have sparked interest in extending the technique to include spatial resolution. In this manner, it is possible to spatially map variables such as elemental composition and micro-hardness in situ. Jolivet et al. have published a comprehensive review of this evolving field [195]. The principle of the scanning LIBS imaging technique is presented in Figure 36.

Laser-induced breakdown spectroscopy was originally a destructive inspection method for determining the chemical composition of the tested samples. In metal AM, specifically in laser-melting based metal AM, laser-induced breakdown spectroscopy was turned into a nondestructive method detecting the plasmonic radiations offered by the fabricating laser in the additive manufacturing process. Without the laser-induced plasmonic radiations, the method cannot be performed. Hence, in solid-state AM processing, laser-induced breakdown spectroscopy cannot be applied without advanced experimental designs. One potential solution for applying laser-induced breakdown spectroscopy in solid-state AM processes can be adding an extra portion on the printing model for chemical composition monitoring only. The additional section can be printed along and together with the printing sample. Laser-induced breakdown spectroscopy can be applied on the other portion with laser assistance for monitoring the abnormal composition formation.

## 7. Other Diagnostic Methods

During the additive manufacturing process, especially for laser powder bed fusion (LPBF), high thermal gradients will cause cyclic expansion and contraction of the printed part, which leads to residual stresses. Researchers have used Raman spectroscopy, a non-destructive light scattering technique, to determine the residual stress of additively manufactured AlSi10Mg [196]. Raman spectroscopy has also been used to determine the crystalline phases and chemical structures of additively manufactured composites that are challenging to manufacture with conventional techniques. For example, copper and diamond [197] or monitoring the oxidation activity for the metal surface [198] can provide molecular-level information and operate over a wide temperature and pressure range in all liquid, solid, and gas phases. The neutron technique is similar to X-ray scattering methods, and the most important difference is the penetration depth. For X-ray, the penetration ranges from a few microns to millimeters, with high energy radiation from the surface, whereas neutrons can penetrate several centimeters. Another difference is that neutrons can provide appreciable scattering signals from light elements. Therefore, neutron scattering can be used as a non-destructive technique for determining the residual stress of additive manufacturing products by measuring the residual strain [199,200]. The listed methods are not commonly available. However, the performance and unique results from these methods are very useful and cannot be substituted by any other technique.

## 8. Conclusions and Discussion

The various ex-situ inspection and in-situ monitoring techniques all provide unique information about samples. For comprehensive research and development, all the above-listed diagnostic techniques can evaluate discontinuities, microstructure, physical properties, and transient conditions during processing. The destructive ex-situ tests were broadly applied in the existing literature to visualize the discontinuities and micro-structures and to test the physical properties. The samples were produced for exchanging information. In this sense, the destructive methods carry a huge amount of information. For example, electron-based imaging provides information about discontinuities and micro-structures, and tensile tests highlight elasticities and plasticities. In the meantime, the destructive methods maintain reliable accuracy. Some non-destructive methods could also observe discontinuities and elasticity, such as several ultrasonic techniques. However, the trade-off in using the non-destructive tests is the decreased resolution and additional uncertainties. The detection resolution of an instantaneous ultrasound phased-array system can reach 100 μm. The electron-based imaging tools can detect higher-resolution features that are one or two magnitudes smaller in size. The ultrasonic methods can only offer a dynamic modulus for evaluating mechanical properties due to the anisotropy in the metal AM products, which might not represent the same low-frequency mechanical response presented by its static modulus. Thus, dependent on the fields to apply the inspection, the destructive test methods offer advantages for academia, and industrial fields would benefit from well-developed non-destructive testing.

The ex-situ or post-processing of the 3D-printed or AM structures hardly provides any information about the transient change in the mechanical behavior or thermokinetics of metallurgical transients. Thereby, the in-situ monitoring techniques represent the only method to observe the transients during the printing process. Some advanced studies have used post-test results to backfit and reproduce the phenomenon with the assistance of numerical simulation. However, generally, this is not easy to achieve. The existing in-situ monitoring applied in metal AM processing mainly involves thermal imaging techniques and ultrasound monitoring, obtaining different types of information. The thermal imaging techniques commonly involve monitoring the melting pool during printing.

On the other hand, ultrasonic monitoring can distinguish the rapid mechanical property variation during the printing process. The current stage of the metal AM monitoring techniques still lacks in-situ methods. As the ex-situ methods show, newly developed in-situ methods would be beneficial to observe other information rather than melting pool condition and rapid property variations, such as discontinuities and residual stress distribution.

Each of the ex-situ inspection and in-situ monitoring methods for metal additive manufacturing provides unique information and advantages. The ex-situ methods are much more broadly applied in metal AM process and product studies. The well-developed destructive testing methods performed excellently on the metal AM products, which are more suitable for research in academia and quick tests in industrial applications. For inspecting final products, destructive testing is not as useful as the non-destructive methodologies outlined in this work. However, non-destructive methods usually have more limitations and uncertainties. Overall, the metal AM field has enough reliable ex-situ techniques available but needs urgent development and investigation of more practical and reliable in-situ monitoring methods in order to obtain more real-time and physical information during the printing process in order to better understand the processing. This would move the field of metal AM processes in a new direction.

## Figures and Tables

**Table 1:** Summary of built features in powder bed and powder injection metal AM techniques.

Features	Powder Bed (LPBF, SLM)	Powder Injection (DED, DMD)	References
Particle size (µm)	15–45	40–110	[13,14]
Environment	Inert environment (Ar/N) inside processing chamber	Inert environment (Ar/N) or inert shielding gas (Ar/N)	[13,14]
Power range (W)	100–400	300–1000	[15,16]
Beam spot (µm)	30–600	660–5000	[15,17]
Scanning speed (mm/s)	300–1200	1–20	[18,19]
Microstructure	Relatively fine-grained structure, non-equilibrium phases. Usually, columnar grains are formed.	Fine-grained structure with near-equilibrium and non-equilibrium phases. Variation of cellular, columnar, and equiaxed grains based on thermokinetics along the build direction.	[14,20]
Three-dimensional defects	Relatively higher porosity and cracking	Lower porosity and cracking	[21,22]
Surface finish	Relatively lower surface roughness	Higher surface roughness (30.6–63.9 µm)	[23,24]
Residual stresses, σ (Mpa)	Relatively higher	Lower	[15,23]
Advantages	Fabrication of complex geometries with optimal material usage. The reusability of powder is higher.	Direct injection of powder at the built point reduces the need for a higher quantity of raw materials. The fabrication rate is relatively higher.	[17,25]
Limitations	Expensive machinery usage with longer building time	Fabrication of complex geometries is challenging, with build parts having considerably high surface roughness.	[17,25]

**Table 2:** Summary of cold spray AM and friction stir AM processes [28,29].

Features	Cold Spray AM	Friction Stir AM
Raw material	Powder particle spray	Feed rod/powder is thermodynamically deformed and deposited
Powder melting	No	No
Feed mode	Direct deposition of powder	Direct deposition of powder
Working mechanism	Powder jet propulsion impact via high-pressure gas (0.5–6 MPa) at 25–1000 °C	Thermo-mechanically imposed solid-state diffusion of feedstock on the substrate via friction stirring of tools at 500–2000 rpm
Microstructure and adhesion between parts	High-pressure gas causing a heavy impact on particles, leading to metallic bonds and the production of thermodynamically stable microstructures	Can produce variable microstructures and is application-specific. Microstructure varies from the core of a layer (coarse) towards the joint of layers(fine).
Mechanical properties of the as-fabricated part	Poor	Excellent at joint areas
Fabrication rate	Low	Relatively high
Build size	Usually confined to coating and cladding. Not explored for larger dimensions.	Can fabricate large parts
Raw material usage	High buy to fly ratio	Judicious usage of raw materials

**Table 3:** Summary of characteristics of fusion-based AM processing and solid–liquid mixed-phase AM processing techniques [30,31,32,33,34,35,36,37].

Techniques	Fusion-Based Laser AM Processes (LPBF/DED)	Solid–Liquid Mixed-Phase AM Techniques (BJP)
Raw materials	Powder	Powder, liquid binder
Layer adhesion	Powder melting and consolidation	Selectively joined by a binder material
Product achieved (as fabricated)	Finished product. Heat treatment is non-mandatory	A green body that requires mandatory sintering or heat treatment
Mechanical properties of as-built parts	Better mechanical properties of the as-built parts	Much weaker as-built parts with inferior properties before sintering
Maximum build envelope	600 × 400 × 500 mm	4000 × 2000 × 1000 mm
Minimum layer thickness	0.03 mm	0.09 m
Minimum feature size	0.04–0.2 mm	0.1 m
Density	Up to 99.9%	-
Build rate	Slow process	Depends on the binder curing time
Economic aspect	Expensive process	Cheaper processes, no requirement for laser or equivalent expensive heat source

**Table 4:** Summary of additive vs. subtractive manufacturing processes [44,45,46,47,48].

Features	Additive Manufacturing Processes	Conventional Manufacturing Processes
Raw material efficiency	AM imparts higher raw material efficiency as much of the left-over materials can be reused after minimal or no post-processing	Conventional manufacturing or removal of a large amount of material from a larger part imparts lower material efficiency
Resource efficiency	AM does not require additional resources and helps in improving supply-chain dynamics	Additional resources are required in conventional processes such as cutting tools, fixtures, etc.
Energy efficiency	The production of raw materials (e.g., powders) for AM can tag AM processing as low-energy-efficiency techniques	Additional high energy consumption for raw material production is not involved in conventional processes
Production flexibility	AM is economical in small-batch set-ups. Helps in synchronizing production with demand	Line balancing bottlenecks often occur in conventional techniques
Part flexibility	Due to no tooling constraints, parts can be fabricated in a single piece	Conventional techniques offer lower part flexibility
Product quality	Depends on the process itself	Depends on the operator skills
Size limitations	Large-sized part production is challenging and requires extended time involvement	Conventional techniques are still the first choice for large part production
Imperfections	AM-produced parts offer higher surface roughness, originating from partially melted or non-melted raw materials	Subtractive techniques offer better perfection in produced parts
Cost	It might require expensive investments	Offers economic advantages if extensive set-ups are required in AM
Environment aspect	AM is environmentally friendly as unused raw materials can be mostly reused	A large quantity of waste material is generated, which might not be so environmentally friendly

**Table 6:** Summary of electron and X-ray combined material characterization techniques [54,55,56,57,58].

Electron and X-ray Combined Analysis Technique	Working Principle	Measuring Process	Measuring Characteristics
Electron Backscattered Diffraction (EBSD)	Reflected secondary or backscattered electrons correspond to lattice diffracting crystal planes	Interaction of incident electron beam with sample surface results in secondary or backscattered electron emission. These emitted electrons may be ejected in Bragg angles and diffract to form Kikuchi bands, corresponding to lattice diffracting crystalline planes.	Crystal orientation, structure, strain, or phase in the material
Energy-Dispersive X-ray Spectroscopy (EDS or EDX)	Electron beam (10–20 keV) interacts with the sample surface and results in the emission of X-rays	Electron beam interaction with sample surface results in the emission of X-rays, which are generated in region ~2 µm in depth	Elemental analysis within a sample
Wavelength Dispersive Spectroscopy (WDS)	Electron beam interaction with the sample surface results in the emission of X-rays	X-rays emitted as a result of electron beam and sample interaction are energy specific to elements that results in their emission	Determination of composition in alloys and mapping secondary phases
X-ray Photoelectron Spectroscopy (XPS)	Surface-sensitive quantitative spectroscopic technique. The sample surface is irradiated with X-rays.	As the sample surface is irradiated with X-rays, monoenergetic photons knock out electrons from atoms in the surface region, and photons with higher energy penetrate deeper into the sample. Spectra are obtained by measuring characteristics of electrons generated in this manner	Chemical state and electronic state of elemental constituents in a sample. It can also detect the valance state of elements.

## Data Availability

Data requests should refer to the original authors this article cited.
